# The spatial synchrony of species richness and its relationship to ecosystem stability

**DOI:** 10.1002/ecy.3486

**Published:** 2021-08-12

**Authors:** Jonathan A. Walter, Lauren G. Shoemaker, Nina K. Lany, Max C. N. Castorani, Samuel B. Fey, Joan C. Dudney, Laureano Gherardi, Cristina Portales‐Reyes, Andrew L. Rypel, Kathryn L. Cottingham, Katharine N. Suding, Daniel C. Reuman, Lauren M. Hallett

**Affiliations:** ^1^ Department of Environmental Sciences University of Virginia Charlottesville Virginia USA; ^2^ Botany Department University of Wyoming Laramie Wyoming USA; ^3^ Department of Forestry Michigan State University East Lansing Michigan USA; ^4^ Department of Biology Reed College Portland Oregon USA; ^5^ Department of Plant Sciences University of California‐Davis Davis California USA; ^6^ School of Life Sciences Arizona State University Tempe Arizona USA; ^7^ Department of Ecology, Evolution, and Behavior University of Minnesota St. Paul Minnesota USA; ^8^ Department of Wildlife, Fish, and Conservation Biology University of California Davis California USA; ^9^ Department of Biological Sciences Dartmouth College Hanover New Hampshire USA; ^10^ Department of Ecology and Evolutionary Biology University of Colorado Boulder Colorado USA; ^11^ Department of Ecology and Evolutionary Biology and Kansas Biological Survey University of Kansas Lawrence Kansas USA; ^12^ Environmental Studies Program and Department of Biology University of Oregon Eugene Oregon USA

**Keywords:** biodiversity, community synchrony, dispersal, ecosystem stability, Moran effect, spatial synchrony

## Abstract

Synchrony is broadly important to population and community dynamics due to its ubiquity and implications for extinction dynamics, system stability, and species diversity. Investigations of synchrony in community ecology have tended to focus on covariance in the abundances of multiple species in a single location. Yet, the importance of regional environmental variation and spatial processes in community dynamics suggests that community properties, such as species richness, could fluctuate synchronously across patches in a metacommunity, in an analog of population spatial synchrony. Here, we test the prevalence of this phenomenon and the conditions under which it may occur using theoretical simulations and empirical data from 20 marine and terrestrial metacommunities. Additionally, given the importance of biodiversity for stability of ecosystem function, we posit that spatial synchrony in species richness is strongly related to stability. Our findings show that metacommunities often exhibit spatial synchrony in species richness. We also found that richness synchrony can be driven by environmental stochasticity and dispersal, two mechanisms of population spatial synchrony. Richness synchrony also depended on community structure, including species evenness and beta diversity. Strikingly, ecosystem stability was more strongly related to richness synchrony than to species richness itself, likely because richness synchrony integrates information about community processes and environmental forcing. Our study highlights a new approach for studying spatiotemporal community dynamics and emphasizes the spatial dimensions of community dynamics and stability.

## Introduction

Synchrony has broad importance in population and community ecology, and recent efforts that integrate perspectives from these subdisciplines have generated new insights into spatiotemporal population and community dynamics (Wang and Loreau 2014, Wilcox et al. 2017, Arribas et al. 2019, Lee et al. 2019, Walter et al. 2021). Population spatial synchrony, where temporal fluctuations in abundance are correlated across populations inhabiting multiple locations, is a fundamental feature of population dynamics observed across taxa and over wide‐ranging spatial scales (Liebhold et al. 2004, Walter et al. 2017). Mechanisms underlying population spatial synchrony include dispersal, spatially correlated environmental fluctuations driving shared demographic responses (Moran effects), and interactions with a species that itself exhibits spatial synchrony (Moran 1953, Liebhold et al. 2004). Spatially synchronous populations are at greater risk of regional extirpation or extinction. This is especially true for species of conservation concern, such as stocks of exploited species (Schindler et al. 2015), as simultaneous rarity reduces the population rescue effect of dispersal (Earn et al. 1998, Heino 1998).

In contrast to population spatial synchrony, community ecology tends to focus on a different kind of synchrony: correlated temporal fluctuations of multiple species' abundances in a single location. This “community synchrony” can alter the stability of its aggregate properties. For example, community synchrony decreases the temporal stability of total abundance or biomass production (Micheli et al. 1999, Loreau and de Mazancourt 2008), which is commonly equated to ecosystem function (Donohue et al. 2016). Alternatively, stability is maintained when species fluctuate independently and especially if their fluctuations negatively covary. This negative covariance between species, commonly known as compensatory dynamics, reflects heterogeneity in species' responses to environmental drivers, possibly mediated through competitive release (Gonzalez and Loreau 2009, Hallett et al. 2017).

As exemplified via the sustained focus on metacommunity theory over the past decade (Leibold et al. 2004, Leibold and Chase 2017), there is growing recognition of the importance of spatial scaling and the interplay of local vs. regional dynamics on community attributes such as biodiversity (De Meester et al. 2016, Shoemaker and Melbourne 2016) and stability (Wang and Loreau 2014, Wang et al. 2019). That many of the factors that are central to population spatial synchrony, including dispersal, temporal environmental variation, and spatial heterogeneity, have also proven important to spatiotemporal community dynamics suggests that we may, a priori, expect that biodiversity (e.g., species richness) could exhibit spatial synchrony, at least under some conditions. To date, however, whether biodiversity commonly exhibits spatial synchrony, and if so, why, is unknown. Here, we focus on spatial synchrony in species richness and explore potential mechanisms through which richness synchrony could arise, as well as its implications.

There are several reasons to investigate synchrony in richness. Biodiversity is often associated with ecosystem function (Tilman and Downing 1994, Schulze and Mooney 2012, Rypel and David 2017) and stability thereof (Cottingham et al. 2001, de Mazancourt et al. 2013). Species richness is widely used to quantify biodiversity, in part because presence‐absence data are more easily obtained than data on abundance, or indices thereof, needed for other measures. Furthermore, studying synchrony in numbers of species bears quantitative similarity to studying synchrony in numbers of individuals, as in population spatial synchrony, even though the generating processes are more complex.

Here, we consider how spatial synchrony in species richness might arise mechanistically. In a given location (e.g., a patch in a metacommunity), fluctuations in richness reflect local colonization and extinction events. Species richness could therefore exhibit spatial synchrony if colonization and extinction dynamics are themselves spatially correlated, for example due to dispersal. Dispersal could in principle produce synchronous fluctuations in species richness even in a competitively neutral, homogeneous environment. Additionally, environmental fluctuations could themselves cause or enhance richness synchrony (Harrison and Quinn 1989), especially in settings where local extinctions are possible. Spatially correlated environmental fluctuations could also synchronize patch‐level richness by altering available niche space (Shoemaker and Melbourne 2016) or shifting the suite of species favored under current conditions (Pitt and Heady 1978). We expect that Moran effects on species richness are likely given that biodiversity can fluctuate in response to climatic variation (Peco et al. 1998), and that Moran effects on populations comprising the community, which are common (Liebhold et al. 2004), may manifest in community metrics.

Drawing on the implications of spatial synchrony for population stability, and the implications of diversity and community synchrony for stability, we predict that spatial synchrony in richness will relate strongly to stability of ecosystem function at the landscape scale. More biodiverse systems may be more stable in the sense of tending to have lower temporal variance in ecosystem function (Cottingham et al. 2001). Synchrony is destabilizing in the same sense because shared fluctuations reinforce each other and thereby total to large variations in the aggregate, while asynchronous fluctuations cancel out (Hallett et al. 2014, Anderson et al. 2021).

This study integrates insights from a theoretical metacommunity model with a synthesis of 20 empirical metacommunities from terrestrial grassland and coastal marine biomes to examine the prevalence of spatial synchrony in species richness, the ecological factors that can promote or diminish it, and how it can provide insight into the stability of ecosystem function. Specifically, we address the following research questions: (1) Do local fluctuations in species richness exhibit spatial synchrony across metacommunity patches? (2) Are the well‐documented drivers of population spatial synchrony (i.e., Moran effects and dispersal) also key drivers of spatial synchrony in richness? (3) Does a community's strength of spatial synchrony of richness relate to ecosystem stability and how does this compare to relationships between richness and stability? Overall, our study demonstrates the commonness of spatial synchrony in species richness, identifies key abiotic and biotic factors that alter the degree of richness synchrony, and explores how the spatial synchrony of richness may be strongly related to the temporal stability of ecosystem function.

## Methods

### Quantifying synchrony in community properties

Although spatial synchrony has mainly been quantified for population variables, spatial synchrony can, in principle, be quantified for any time‐varying quantity with measurements taken through time in different places. We measured spatial synchrony of species richness as follows. We began with data consisting of species' abundances at *P* locations (hereafter, patches) through time. We measured species richness of each patch at each time step to compute richness, *R_p_*_,_ *_t_*, where *p* is the patch and *t* is the time‐step. We then linearly detrended the time series, standardized variances of each time series to one, and computed the matrix of Spearman correlations for fluctuations in richness through time between all patch pairs. Finally, the lower triangle (excluding the diagonal) of the correlation matrix was averaged to produce one representative value for each site (metacommunity), as commonly occurs when examining community synchrony (Kent et al. 2007, Hallett et al. 2014), and allows us to compare across metacommunities.

### Theoretical modeling

To examine when we expect to observe spatial synchrony of richness and what mechanisms most alter it, we applied the above workflow to simulated metacommunities. Coupling a theoretical model that incorporates known underlying mechanisms with a statistical analysis of the spatial synchrony of richness provides insight into the behavior of synchrony under different ecological mechanisms. In brief, our metacommunity model connects local patch‐level dynamics to regional dynamics via dispersal. Growth, competition, and environmental effects occur within a patch, environmental conditions of each patch vary both through space and time, and patches are connected via dispersal of individuals. Within‐patch dynamics follow a multispecies, metacommunity extension of the model of Loreau and de Mazancourt (2013), which is a discrete‐time modification of classic Lotka‐Volterra competition dynamics that incorporates both demographic and environmental stochasticity and disentangles species' carrying capacities from their competitive effects (Loreau and de Mazancourt 2008, Loreau 2010).

First, prior to local population dynamics, dispersal between patches occurs. We model dispersal as both local and global (global results are presented in Appendix S1). Abundance *N* of each species *s* in a given patch *p* after dispersal, but before population growth, is indexed as time step *t* + δ, and is modeled as

(1) $$N_{s,p,t+\delta }=N_{s,p,t}‐E_{s,p,t}+I_{s,p,t}$$

where *E_s_*_,_ *_p_*_,_ *_t_* denotes emigration of species *s* from patch *p* while *I_s_*_,_ *_p_*_,_ *_t_* denotes immigration. For global dispersal, *E_s_*_,_ *_p_*_,_ *_t_* = −*d_s_N_s_*_,_ *_p_*_,_ *_t_* and

$$I_{s,p,t}=d_{s}\sum_{x\neq p}\frac{N_{s,x,t}}{P‐1}.$$

where *P* denotes the total number of patches in the metacommunity, and *d* is the across‐patch stochastic dispersal probability, where propagule dispersal is binomially distributed with the probability of success equal to *d* (Shoemaker and Melbourne 2016). Alternatively, for local dispersal, propagules disperse only to their nearest neighbor patches, and the landscape is modeled as a square lattice with wrap‐around boundaries (Kerr et al. 2002).

Following dispersal, within a patch, *p*, the abundance of each species changes through time *t* according to:

(2) $$N_{s,p,t+1}=N_{s,p,t+\delta }\exp[r_{s}\left(1‐\frac{N_{s,p,t+\delta }}{K_{s}}‐\sum_{j\neq s}\frac{\beta_{s,j}N_{j,p,t+\delta }}{K_{j}}\right)+\sigma_{e,s}\mu_{e,p,t}+\frac{\sigma_{d,s}\mu_{d,s,p,t}}{\sqrt{N_{s,p,t+\delta }}} \backslash Bigg].$$

In the above equation, *r* is a species' intrinsic (density‐independent growth rate), *K* is its carrying capacity in a patch, and β*_s_*_,_ *_j_* is the competition coefficient of species *j* on species *s*. Compared to a classic Lotka‐Volterra model, here we separate species' interspecific competitive effects (β*_s_*_,_ *_j_*) from their carrying capacities (*K_s_*). This formalization is related to the α coefficients of Lotka‐Volterra dynamics where β*_s_*_,_ *_j_* = α*_s_*_,_ *_j_K_j_*/*K_s_* (Loreau and de Mazancourt 2013). Model parameters and their values are given in Table 1.

**Table 1:** Model parameters, description, and ranges used in generating simulations.

Parameter	Description	Value/Range
*S*	number of species in the regional species pool	sample (min = 15, max = 55)
*P*	number of patches in the metacommunity	sample (min = 9, max = 49)
*h*	spatial heterogeneity between patches	uniform (min = 0, max = 0.5)
*a*	temporal autocorrelation in climate	uniform (min = 0, max = 0.75)
*b*	magnitude of the effect of climate	(1−*a* ^2^) 0.5
µ*_e_*_,_ *_p_*_,_ *_t_*	environmental fluctuations in each patch	normal (mean = *c_t_*, sd = *h*)
env_sd_	standard deviation of effect of env. variation	uniform (min = 0.05, max = 0.5)
σ*_e_*_,_ *_s_*	response of each species to environmental variation	normal(mean = 0, sd = env_sd_)
µ*_d_*_,_ *_s_*_,_ *_p_*_,_ *_t_*	demographic fluctuations	normal(mean = 0, sd = 1)
σ*_d_*_,_ *_s_*	effect of demographic fluctuations	uniform (min = 0, max = 0.75)
*r*_avg_	scaled average growth rate	uniform (min = 0, max = 0.25)
*r_i_*	species‐specific growth rate	uniform (min = 0.5 − *r*_avg_, max = 0.5 + *r*_avg_)
β_max_	maximum competition coefficient	uniform (min = 0, max = 0.5)
β*_s_*_,_ *_j_*	competition coefficient of species *j* on species *s*	uniform (min = 0, max = β_max_)
*d*	dispersal rate	uniform (min = 0, max = 0.2)
*K_s_*	carrying capacity	lognormal (logmean = 3, logsd = 1)

Note

Abbreviations min and max stand for minimum and maximum, respectively.

Demographic stochasticity is incorporated as a traditional first‐order normal approximation, and represents inherent variation between individuals in birth and death rates (Lande et al. 2003). Here, σ*_d_*_,_ *_s_* is the susceptibility of species *s* to demographic fluctuations and µ*_d_*_,_ *_s_*_,_ *_p_*_,_ *_t_* are independent, identically distributed normal variables with mean zero and variance one representing fluctuations through time for each species in each patch.

Environmental stochasticity is similarly incorporated through µ*_e_*_,_ *_p_*_,_ *_t_*, which represents environmental variation in each patch through time and σ*_e_*_,_ *_s_*, which quantifies the impact of environmental variation on each species *s*. While Loreau and de Mazancourt (2013) restricted µ*_e_*_,_ *_p_*_,_ *_t_* to be uncorrelated, here we extend their model to allow for temporal autocorrelation in environmental conditions and variation across patches. To do so, we follow the formulation from Ripa and Lundberg (1996), where we first create a time series of regional climate conditions, *c*

(3) $$c_{t+1}=ac_{t}+b\phi_{t}.$$

We set the initial condition *c*_0_ = 0. In Eq. 3, *a* controls the temporal autocorrelation of the climate where *a* = 0 represents uncorrelated, white noise. When *a* > 0, successive events are more likely to be similar to other events that occur closely in time (Ripa and Lundberg 1996). Stochastic noise ϕ*_t_* ˜ Normal(0,1) is scaled by the magnitude of its effect, *b*. Following Ripa and Lundberg (1996), *b* = (1−*a* ^2^)^0.5^, which restricts var(*c*) to be the same for all autocorrelation (*a* values) considered. From the time series of regional climactic conditions, we create between‐patch variation that represents the degree of microhabitat variation, assuming that spatial heterogeneity is less than temporal variation to match the spatial scale of our empirical metacommunities (Gómez‐Aparicio et al. 2005, Ford et al. 2013). To create microhabitat variation, µ*_e_*_,_ *_p_*_,_ *_t_* ˜ Normal(*c_t_*,*h*) where *h* controls the variability between patches.

Using the above model, we examine the relative effects of multiple abiotic and biotic factors on the spatial synchrony of richness. We simulated metacommunities that differed in: richness of the regional species pool (*S*; matching the empirically observed range), number of patches (*P*; again matching the empirically observed range), spatial heterogeneity in patch quality (*h*), temporal autocorrelation of the regional climate conditions (*a*), species' responses to environmental fluctuations (σ*_e_*_,_ *_s_*), species' growth rates (*r*), species' competitive strengths (β*_s_*_,_ *_j_*), and dispersal rates (*d*). All variable parameters were drawn independently from the distributions in Table 1, which also includes values for non‐focal parameters (e.g., µ*_d_*_,_ *_s_*, *K_s_*). We began each simulation with species' abundances set to their carrying capacities, *K_s_*, and as the model quickly settles on its steady‐state distribution, we simulated our model for 100 time steps. We used the first 50 time steps as a “burn‐in” period to remove any effect of initial conditions on our analyses. The last 50 time steps were used for calculating spatial synchrony of species richness, creating time series for each simulation with length on the same order as those from our empirical analyses. We ran a total of 2,500 simulations and calculated spatial synchrony in species richness and the coefficient of variation in total abundance in all simulations.

### Empirical data sets

We paired our theoretical model with a study of 20 empirical metacommunities encompassing both grassland and coastal marine habitats, primarily drawing from the United States Long Term Ecological Research Network. All data sets consisted of regularly sampled observations of species' abundance in a community for at least six plots and 10 yr (Table 2). All data sets focused on primary producer taxa in unmanipulated plots. Plots in empirical data sets were taken to be equivalent to patches and for consistency are called patches henceforth. At some sites, up to three distinct metacommunities were considered separately. Metacommunities were considered distinct on the basis of diverging habitat such as soil type or disturbance frequency, dissimilarity in species present, and the opinion of investigators familiar with these sites. Additional description of data set properties and provenance is provided in Appendix S1: Section 1. We included all species having non‐zero abundance in at least 5% of patch‐by‐time combinations in order to minimize any potential bias of observational error on our results. Preliminary analyses using different thresholds from 0% (no threshold) to 10% indicated that measured spatial synchrony of richness was robust to our 5% threshold choice.

**Table 2:** Empirical data sets.

Data set	Year	Length (yr)	*N*_plots_	Extent (km)	Biome	*N*_taxa_	Variable	Plot size (m^2^)
DRT	2005	11	6	16.5	marine	25	percent cover	0.2
HAY	1943	30	13	0.05	grassland	16	percent cover	1
JRG	1983	34	12	0.03	grassland	25	percent cover	1
JRN_BASN	1989	24	49	0.09	grassland	44	biomass	1
JRN_IBPE	1989	24	49	0.08	grassland	51	biomass	1
JRN_SUMM	1989	24	49	0.09	grassland	53	biomass	1
KNZ_UP	1983	33	20	0.17	grassland	47	percent cover	10
KNZ_LOW	1983	33	20	0.23	grassland	44	percent cover	10
LOK	1996	20	14	49.0	marine	28	percent cover	0.25
MAU	2001	16	9	50.4	marine	21	percent cover	0.25
MCR_BACK	2006	10	30	16.65	marine	15	percent cover	0.25
MCR_FRNG	2006	10	30	15.67	marine	28	percent cover	0.25
MCR_OUT	2006	10	30	17.29	marine	25	percent cover	0.25
MDK	1996	20	8	55.4	marine	24	percent cover	0.25
SBC	2001	18	34	73.38	marine	30	biomass	80
SEV_B	2002	13	30	0.70	grassland	42	biomass	1
SEV_C	1999	16	30	1.33	grassland	29	biomass	1
SEV_G	1999	16	22	0.81	grassland	27	biomass	1
UPK	1996	20	10	44.7	marine	23	percent cover	0.25
USVI	1992	26	6	1.38	marine	17	percent cover	0.25

Notes

Data set codes correspond to, respectively, DRT, Dry Tortugas, Florida; HAY, Hayes, Kansas; JRG, Jasper Ridge, California; JRN_BASN, Jornada LTER Basin; JRN_IBPE Jornada LTER International Biological Program exclosure; JRN_SUMM Jornada LTER Mount Summerford; KNZ_UP, Konza Prairie upland; KNZ_LOW, Konza Prairie lowland; LOK, Lower Florida Keys; MAU, Maui, Hawaii; MCR_BACK, Moorea Coral Reef LTER Backreef; MCR_FRNG, Moorea Coral Reef LTER fringing reef; MCR_OUT, Moorea Coral Reef outer reef; MDK, Middle Florida Keys; SBC, Santa Barbara Coastal LTER; SEV_B, Sevilleta LTER blue gramma; SEV_C, Sevilleta LTER creosotebush; SEV_G, Sevilleta LTER black gramma; UPK, Upper Florida Keys; USVI, U.S. Virgin Islands LTER. Year corresponds to the initial year of the time series. Extent gives the maximum interpatch distance, in km. *N*_taxa_ gives the total number of taxa (i.e., γ‐diversity) of the metacommunity.

### Analyses of empirical and theoretical communities

We applied parallel analyses to our model simulations and empirical data to address our research questions. We first asked whether species richness exhibits spatial synchrony (Q1). To address this question using theoretical simulations, we computed the mean richness synchrony for all 2,500 simulated metacommunities and examined the distribution of theoretical richness synchrony measures. To address this question empirically, we computed the mean spatial synchrony of richness for all 20 focal metacommunity data sets and tested the statistical significance of spatial synchrony of richness for each. Significance testing was performed by comparing empirical values to surrogate values from simulated data generated under a null hypothesis of no spatial synchrony, while preserving the temporal autocorrelation structures of the empirical data. Surrogate data sets were generated by taking the amplitude‐adjusted Fourier transform of input species richness time series, randomizing the phases of the Fourier components so that any remaining spatial synchrony is due to chance alone, inverse transforming the data, and measuring the synchrony of the surrogates (Schreiber and Schmitz 2000). We generated 1,000 surrogates for each data set, and considered richness synchrony statistically significant when the empirical value exceeded 95% of surrogates.

To determine the key drivers of spatial synchrony in richness (Q2), we used multiple linear regression to measure the combined effects of multiple predictors on the synchrony of richness. Predictors were re‐scaled to have a mean of zero and standard deviation of 1 so that regression coefficients corresponded to effect sizes. In our theoretical simulations, we examined the effects of key parameters that fall into three general categories: abiotic temporal factors, abiotic spatial factors, and demographic factors. Abiotic temporal factors included in our regression are the effect of environmental variation on species (env_sd_, the variability of environmental driver σ*_e_*), and temporal autocorrelation in environmental variation (*a*) (Table 1). Abiotic spatial factors include the total number of patches (*P*) and the amount of patch heterogeneity (*h*). Finally, we examined the effect of demographic variation, specifically in the parameters: average species' density‐independent growth rates (*r*_avg_), maximum competitive strength (β_max_), and species' dispersal rates (*d_s_*).

To answer Q2 for empirical metacommunities, we considered the following predictor variables: biome (terrestrial or marine), metacommunity extent (maximum distance between patches), species richness, evenness, beta diversity, and species turnover rate. To facilitate model‐data comparisons, we also examined the effects of species richness, evenness, beta diversity, and turnover rate in simulated metacommunities. Species richness and evenness were the mean richness and evenness of individual patches, averaged across time. Spatial beta diversity was the mean Jaccard similarity (Hallett et al. 2016) among patches, with the species list for each patch inclusive of all years in the time series (after removing species present in <5% of patch‐years). Turnover rate was the average patch‐level temporal turnover in species composition (Hallett et al. 2016), and metacommunity extent was the maximum distance between patches, measured in kilometers.

To address whether the strength of synchrony in richness predicts ecosystem stability (Q3), we measured the temporal stability of ecosystem function as −1× the coefficient of variation (CV) over time of metacommunity total biomass/cover as a measure of ecosystem stability. That is, $‐1\times \left(\hat{\sigma }/\hat{\mu }\right)$, where $\hat{\mu }$ is the sample mean and $\hat{\sigma }$ is the sample standard deviation. We multiplied values by −1 so that increases in the statistic corresponded to increases in stability. Other studies have used 1/CV, but in our data this created skewed distributions. We examined how richness synchrony predicts ecosystem stability using linear regression, and compared the strength of this relationship to the relationship between ecosystem stability and: species richness, evenness, beta diversity, and turnover rate. We focus primarily on the often‐studied relationship between richness and ecosystem stability (e.g., Tilman and Downing 1994, García‐Palacios et al. 2018). Here, species richness is the average richness over all patches and time steps (years).

## Results

**Fig. 1:**
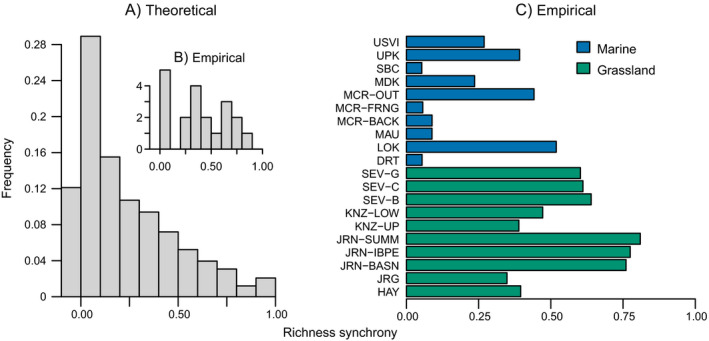
Spatial synchrony in species richness in (A) 2,500 simulated and (B, C) 20 empirical metacommunities.

In both our theoretical model and across 20 empirical metacommunities, spatial synchrony in species richness varied widely among communities, spanning nearly the entire plausible range of the statistic (Fig. 1). The distributions of theoretical and empirical richness synchrony were qualitatively similar (Fig. 1a, b). Coastal marine metacommunities tended to exhibit less richness synchrony than terrestrial grasslands, but also tended to have the larger spatial extents (Table 2). The magnitudes of spatial synchrony in richness tended to be significantly greater than surrogates representing a null hypothesis of no synchrony, suggesting that spatial synchrony of richness is a common phenomenon across ecosystems (Appendix S1: Section 2); in all empirical metacommunities, *P* < 0.05, with the exception of Dry Tortugas (Florida Keys) corals (DRT; *P* = 0.18) and Maui, Hawaii corals (MAU; *P* = 0.052).

**Fig. 2:**
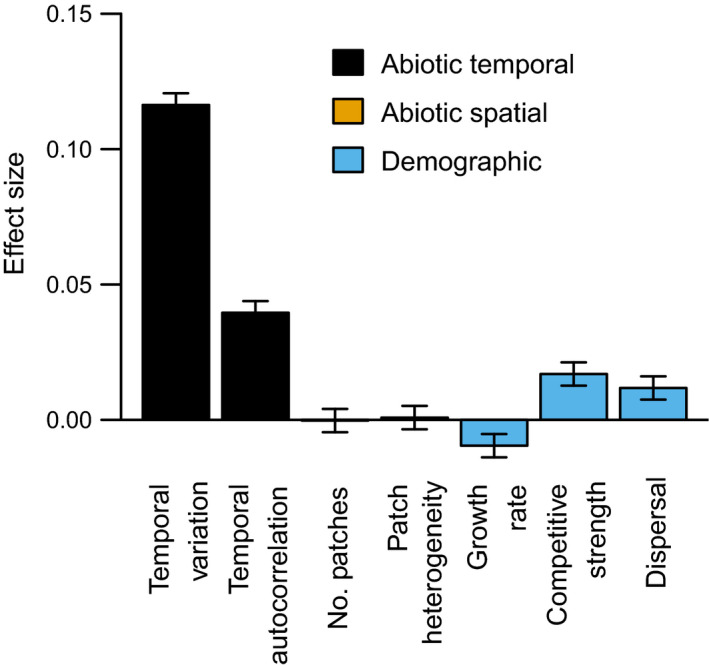
Effect sizes of variation in model parameters on the degree of spatial synchrony of richness in simulated metacommunities. Effect sizes are linear regression coefficients on standardized predictors. Error bars indicate 1 standard error.

When examining which parameters predominantly alter the synchrony of richness in our model, we found that temporal abiotic variation had the strongest effect, followed by demographic rates. Specifically, the effect sizes indicated that the strength of temporal environmental variation (env_sd_) and the degree of autocorrelation in the temporal environmental fluctuations (*a*) had the strongest effects on richness synchrony (Fig. 2). Dispersal (*d*) and competitive strength (β_max_) had smaller, but still positive effect on richness synchrony. The positive effect of dispersal was consistent with our expectations from population synchrony, where increasing dispersal increases population synchrony. Surprisingly, however, spatial heterogeneity in environmental variation had essentially no effect on richness synchrony. This combination of predictors explained 25% of variation in richness synchrony across 2,500 simulations.

**Fig. 3:**
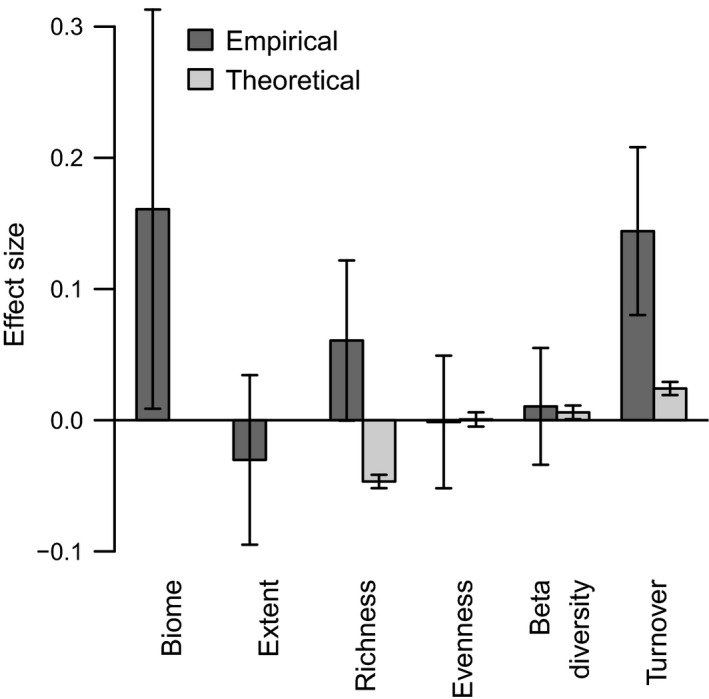
Effect sizes of variation in attributes of empirical and theoretical metacommunities on spatial synchrony of richness. Effect sizes are linear regression coefficients on standardized predictors. There is no direct analog of biome or extent in our theoretical simulations, so no bar is drawn. Error bars indicate 1 standard error.

In empirical metacommunities, biome (i.e., marine vs. grassland ecosystems) was strongly related to richness synchrony, but with a large standard error (Fig. 3). Because both the degree of spatial autocorrelation in environmental conditions and the rate of dispersal between patches typically decrease as the distance between them grows, we expected that extent would have a negative effect on richness synchrony, consistent with dispersal and Moran effects acting as key drivers of richness synchrony. Consistent with our prediction, metacommunity extent was negatively related to synchrony in richness, however with a large standard error (Fig. 3).

As some underlying biological and abiotic factors were impossible to measure in observational studies, we examined potential covariates of richness synchrony that were calculated for both theoretical models and observational data. There was a strong positive relationship between species turnover on richness synchrony across both theoretical and empirical metacommunities (Fig. 3). This is consistent with the fact that changes in species richness imply turnover, but also highlights how community structure and environmental perturbations also likely shaped the spatial synchrony of richness since these factors influence turnover rates (Kraft et al. 2011, Myers et al. 2015). Given that some communities may be more prone to turnover than others when faced with environmental variation, communities may vary in the magnitude of spatial synchrony of richness. In empirical communities, richness synchrony was positively related to the average richness of the metacommunity, but the standard error was large; in theoretical metacommunities, the effect had a similar magnitude but was negative (Fig. 3). In both theoretical and empirical metacommunities there was no substantial effect of beta diversity on richness synchrony. For theoretical metacommunities only, we further examined the importance of beta diversity using the decomposition method of Baselga and Orme (2012) into components associated with change in species number vs. species replacement between communities. The component associated with change in species number had a positive effect on richness synchrony and the component associated with species replacement had a negative effect on richness synchrony. We did not examine this for empirical metacommunities because of the much lower sample size. Neither model nor data show a notable effect of evenness on richness synchrony. In our simulations, these possible explanatory variables were emergent properties of underlying community assembly mechanisms, not directly controlled. This combination of predictors explained 69% of variability in richness synchrony in empirical metacommunities, and 5% of variability in richness synchrony in simulated metacommunities.

**Fig. 4:**
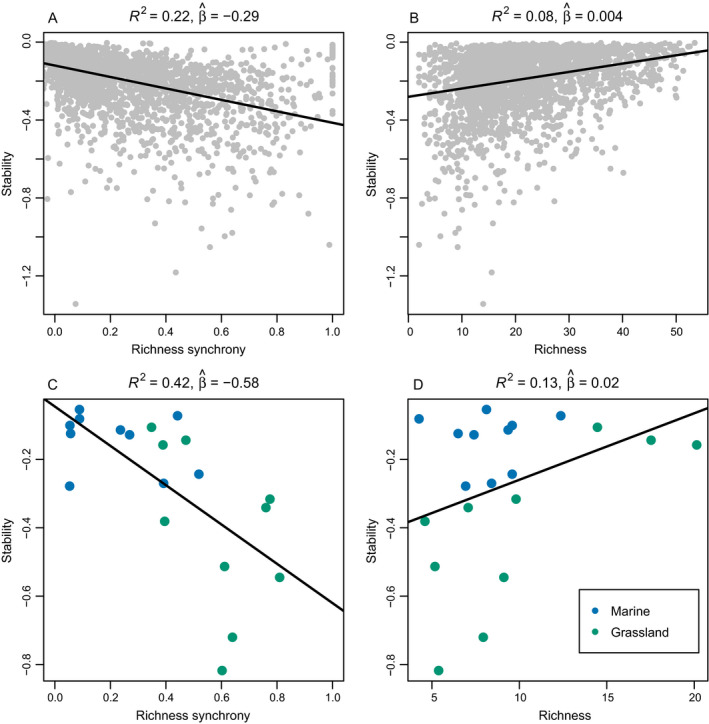
Richness synchrony is related to stability of ecosystem function in (A) theoretical and (C) empirical metacommunities, and more strongly so than species richness itself in both (B) theoretical and (D) empirical metacommunities. Stability is measured, for simulations, as the −1× the coefficient of variation (CV) of total abundance, and for empirical data sets as that of total biomass or total cover, depending on units of the underlying data (Table 2).

Importantly, spatial synchrony of richness was negatively related to the stability of ecosystem function in both theoretical and empirical metacommunities, and exhibited a stronger relationship with stability than species richness itself (Fig. 4). Both theoretical and empirical relationships between the spatial synchrony of richness and community stability were relatively strong (*R* ^2^ = 0.22 and *R* ^2^ = 0.42, respectively), compared to the relationship between diversity and stability (*R* ^2^ = 0.08 and *R* ^2^ = 0.13, respectively). As such, across metacommunities and underlying mechanisms, as manipulated in our simulation modeling, the spatial synchrony of richness emerged as the stronger predictor of stability. Additionally, the spatial synchrony of richness was generally more strongly related to stability than evenness, beta diversity, turnover rate, although the relationship with turnover had an approximately equal *R* ^2^ as for richness synchrony (Appendix S1: Section 3).

Theoretical simulations using global vs. local dispersal yielded consistent results (Appendix S1: Section 4).

## Discussion

Metacommunities often exhibit spatially synchronous fluctuations in species richness (Q1) that are driven in part by Moran effects and dispersal (Q2), two canonical drivers of population spatial synchrony (Moran 1953, Liebhold et al. 2004, Walter et al. 2017). In both mathematical models and observational data spanning marine and terrestrial metacommunities, spatial synchrony of richness was negatively correlated with ecosystem stability, and showed a stronger correlation than species richness itself (Q3). These findings integrate perspectives on spatial synchrony from population ecology with biodiversity's implications for ecosystem stability and function, and reinforce the importance of spatial dimensions of stability (Wang and Loreau 2014, Wilcox et al. 2017, Lamy et al. 2019, Gonzalez et al. 2020, Wang et al. 2019).

Spatial synchrony in species richness appears to be a common phenomenon. Across 20 empirical metacommunities in grassland and coastal marine habitats, spatial synchrony in richness varied substantially, but in 90% of cases was greater than expected under a null hypothesis of no spatial synchrony. In addition, spatial synchrony in species richness has been documented in two recent studies (Arribas et al. 2019, Barringer et al. 2020), but these studies considered only a few empirical metacommunities. In our study, terrestrial ecosystems tended to exhibit higher spatial synchrony in species richness. Marine metacommunities tended to have larger spatial extents (Table 2), which may partially explain this pattern due to the potential for decreased dispersal and environmental spatial correlation with increasing spatial extent. The biomes also tended to differ in the typical lifespans of individuals in the community (e.g., long‐lived corals vs. a mix of annual and perennial plants), possibly affecting the sensitivity of the community to interannual environmental variability.

The variability in the degree of spatial synchrony of richness exhibited by a metacommunity was influenced by attributes of the environment, especially the degree of temporal variability in environmental conditions, and by the structure of the community. Fluctuations in species richness imply year‐to‐year species turnover, and some communities will be more prone to turnover than others due to underlying environmental conditions, disturbance events (Worm and Duffy 2003, Myers et al. 2015), and the demography of constituent species (Ripa and Lundberg 1996, Adler and Drake 2008). How demography alters richness synchrony likely interacts with the nature of environmental fluctuations. Some communities with many rare, extinction‐prone species could exhibit little richness synchrony if extinctions are spatiotemporally random, e.g., if they arise more so from demographic stochasticity than from environmental forcing. By contrast, a community with lower turnover might exhibit greater synchrony in richness if turnover is closely tied to large, spatially synchronous environmental perturbations that locally extirpate, or facilitate the emergence of, multiple species simultaneously.

In fact, the dependence of richness synchrony on both environmental variation and community structure seems to explain small discrepancies between our theoretical and empirical results. In particular, species richness had opposing relationships with richness synchrony in empirical vs. theoretical cases (Fig. 3). In empirical metacommunities, turnover was higher than simulated communities, and richness and evenness were positively correlated, suggesting that as we added more species the aggregated community‐level carrying capacity was partitioned among more species; this lowered abundances on average, making more species susceptible to environmental perturbation and leading to synchronous fluctuations in richness. Meanwhile, in our simulated metacommunities, turnover rates were low and evenness was high but negatively correlated with richness. In this case, higher richness yielded more rare species that tended to stochastically and asynchronously become locally extinct and/or colonize new patches.

The relationship between biodiversity and stability of ecosystem function has generated a great deal of interest in ecology over multiple decades of research (Tilman and Downing 1994, Cottingham et al. 2001, Schulze and Mooney 2012, de Mazancourt et al. 2013). We found that spatial synchrony in richness was more strongly related to stability of total biomass production than was species richness itself (Fig. 4). The negative relationship between richness synchrony and ecosystem stability was expected due to the known destabilizing effects of synchrony in population spatial (Anderson et al. 2021) and community (de Mazancourt et al. 2013, Hallett et al. 2014) synchrony. However, it remains noteworthy since the relationship between synchrony in species number and aggregate abundance (as in this study) is less direct than the relationship between abundances in component units and aggregate abundance (as in population spatial and community synchrony studies). The relative success of the spatial synchrony of richness in predicting ecosystem stability seems to arise primarily because it is a metric that simultaneously reflects information both about community structure and both spatial and temporal environmental variability. For example, greater stability and lower richness synchrony in marine metacommunities, which tended to have larger extents in our study, could reflect spatial insurance effects (Wang and Loreau 2014, Lamy et al. 2019). Our study suggests that richness synchrony may generally be closely related to ecosystem stability and function, providing additional insight into the relationship between biodiversity, synchrony, and stability.

Studying the spatial synchrony of species richness represents a promising approach for investigating drivers of community variability and their consequences for stability of ecosystem function. Although the causes of spatial synchrony in species richness appear complex and remain only partly understood, richness synchrony appears to be an effective integrator of several processes linking biodiversity and stability. While investigations of the spatial synchrony of community variables are uncommon now, the growing availability of long‐term, spatially replicated community data sets enables broader application of this approach. Regardless of whether this approach ultimately earns widespread use, the apparent commonness of richness synchrony and its relationship to stability underscore the importance of spatial structure and spatial scale to ecological stability and biodiversity–ecosystem‐function relationships (Chase and Ryberg 2004, Downing et al. 2008, Wang and Loreau 2014, Gonzalez et al. 2020).

## Supporting information

Appendix S1

## Data Availability

Data are publicly available in the Environmental Data Initiative (EDI) repository at https://doi.org/10.6073/pasta/edf22d17ca46d3d1d46fd0b551784eee (grasslands) and https://doi.org/10.6073/pasta/6925bc1b11832a95614bbe9a64bc8d3a (marine). R code for simulations and analyses is archived on Zenodo (https://doi.org/10.5281/zenodo.4786301).

## References

[ecy3486-bib-0001] Adler, P. B. , and J. M. Drake . 2008. Environmental variation, stochastic extinction, and competitive coexistence. American Naturalist 172:E186–E195. 10.1086/591678 18817458

[ecy3486-bib-0002] Anderson, T. L. , L. W. Sheppard , J. A. Walter , R. E. Rolley , and D. C. Reuman . 2021. Synchronous effects produce cycles in deer populations and deer‐vehicle collisions. Ecology Letters 24:337–347. 33314559 10.1111/ele.13650

[ecy3486-bib-0003] Arribas, L. P. , J. L. Gutiérrez , M. Bagur , S. A. Soria , P. E. Penchaszadeh , and M. G. Palomo . 2019. Variation in aggregate descriptors of rocky shore communities: a test of synchrony across spatial scales. Marine Biology 166:44.

[ecy3486-bib-0004] Barringer, B. C. , W. D. Koenig , I. S. Pearse , and J. M. H. Knops . 2020. Population ecology and spatial synchrony in the abundance of leaf gall wasps within and among populations of valley oak (*Quercus lobata*). Population Ecology 62:220–232.

[ecy3486-bib-0005] Baselga, A. , and C. D. L. Orme . 2012. betapart: an r package for the study of beta diversity. Methods in Ecology and Evolution 3:808–812.

[ecy3486-bib-0006] Chase, J. M. , and W. A. Ryberg . 2004. Connectivity, scale‐dependence, and the productivity‐diversity relationship. Ecology Letters 7:676–683.

[ecy3486-bib-0007] Cottingham, K. L. , B. L. Brown , and J. T. Lennon . 2001. Biodiversity may regulate the temporal variability of ecological systems. Ecology Letters 4:72–85.

[ecy3486-bib-0008] de Mazancourt, C. , et al. 2013. Predicting ecosystem stability from community composition and biodiversity. Ecology Letters 16:617–625. 23438189 10.1111/ele.12088

[ecy3486-bib-0009] De Meester, L. , J. Vanoverbeke , L. J. Kilsdonk , and M. C. Urban . 2016. Evolving perspectives on monopolization and priority effects. Trends in Ecology & Evolution 31:136–146. 26778169 10.1016/j.tree.2015.12.009

[ecy3486-bib-0010] Donohue, I. , et al. 2016. Navigating the complexity of ecological stability. Ecology Letters 19:1172–1185. 27432641 10.1111/ele.12648

[ecy3486-bib-0011] Downing, A. L. , B. L. Brown , E. M. Perrin , T. H. Keitt , and M. A. Leibold . 2008. Environmental fluctuations indice scale‐dependent compensation and increase stability in plankton ecosystems. Ecology 89:3204–3214. 31766790 10.1890/07-1652.1

[ecy3486-bib-0012] Earn, D. J. D. , P. Rohani , and B. T. Grenfell . 1998. Persistence, chaos and synchrony in ecology and epidemiology. Proceedings of the Royal Society B 265:7–10. 9470213 10.1098/rspb.1998.0256 PMC1688758

[ecy3486-bib-0013] Ford, K. R. , A. K. Ettinger , J. D. Lundquist , M. S. Raleigh , and J. H. R. Lambers . 2013. Spatial heterogeneity in ecologically important climate variables at coarse and fine scales in a high‐snow mountain landscape. PLoS ONE 8:e65008. 23762277 10.1371/journal.pone.0065008 PMC3676384

[ecy3486-bib-0014] García‐Palacios, P. , N. Gross , J. Gaitán , and F. T. Maestre . 2018. Climate mediates the biodiversity–ecosystem stability relationship globally. Proceedings of the National Academy of Sciences USA 115:8400–8405. 10.1073/pnas.1800425115 PMC6099882 30061405

[ecy3486-bib-0015] Gómez‐Aparicio, L. , J. M. Gómez , and R. Zamora . 2005. Microhabitats shift rank in suitability for seedling establishment depending on habitat type and climate. Journal of Ecology 93:1194–1202.

[ecy3486-bib-0016] Gonzalez, A. , et al. 2020. Scaling‐up biodiversity‐ecosystem functioning research. Ecology Letters 23:757–776. 31997566 10.1111/ele.13456 PMC7497049

[ecy3486-bib-0017] Gonzalez, A. , and M. Loreau . 2009. The causes and consequences of compensatory dynamics in ecological communities. Annual Review of Ecology Evolution and Systematics 40:393–414.

[ecy3486-bib-0018] Hallett, L. M. , et al. 2014. Biotic mechanisms of community stability shift along a precipitation gradient. Ecology 95:1693–1700. 25039233 10.1890/13-0895.1

[ecy3486-bib-0019] Hallett, L. M. , S. K. Jones , A. A. M. MacDonald , M. B. Jones , D. F. B. Flynn , J. Ripplinger , P. Slaughter , C. Gries , and S. L. Collins . 2016. Codyn: an R package of community dynamics metrics. Methods in Ecology and Evolution 7:1146–1151.

[ecy3486-bib-0020] Hallett, L. M. , C. Stein , and K. N. Suding . 2017. Functional diversity increases ecological stability in a grazed grassland. Oecologia 183:831–840. 28097426 10.1007/s00442-016-3802-3

[ecy3486-bib-0021] Harrison, S. , and J. F. Quinn . 1989. Correlated environments and the persistence of metapopulations. Oikos 56:293–298.

[ecy3486-bib-0022] Heino, M. 1998. Noise colour, synchrony and extinctions in spatially structured populations. Oikos 83:368–375.

[ecy3486-bib-0023] Kent, A. D. , A. C. Yannarell , J. A. Rusak , E. W. Triplett , and K. D. McMahon . 2007. Synchrony in aquatic microbial community dynamics. ISME Journal 1:38. 18043612 10.1038/ismej.2007.6

[ecy3486-bib-0024] Kerr, B. , M. A. Riley , M. W. Feldman , and B. J. Bohannan . 2002. Local dispersal promotes biodiversity in a real‐life game of rock–paper–scissors. Nature 418:171–174. 12110887 10.1038/nature00823

[ecy3486-bib-0025] Kraft, N. J. , et al. 2011. Disentangling the drivers of β diversity along latitudinal and elevational gradients. Science 333:1755–1758. 21940897 10.1126/science.1208584

[ecy3486-bib-0026] Lamy, T. , S. Wang , D. Renard , K. D. Lafferty , D. C. Reed , and R. J. Miller . 2019. Species insurance trumps spatial insurance in stabilizing biomass of a marine macroalgal metacommunity. Ecology 100:e02719. 31081945 10.1002/ecy.2719

[ecy3486-bib-0027] Lande, R. , S. Engen , and B.‐E. Saether . 2003. Stochastic population dynamics in ecology and conservation. Oxford University Press on Demand, Oxford, UK.

[ecy3486-bib-0028] Lee, A. M. , B.‐E. Saether , and S. Engen . 2019. Spatial covariation of competing species in a fluctuating environment. Ecology 101:e02901. 31578713 10.1002/ecy.2901

[ecy3486-bib-0029] Leibold, M. A. , et al. 2004. The metacommunity concept: a framework for multi‐scale community ecology. Ecology Letters 7:601–613.

[ecy3486-bib-0030] Leibold, M. A. , and J. M. Chase . 2017. Metacommunity ecology. Volume 59. Princeton University Press, Princeton, New Jersey, USA.

[ecy3486-bib-0031] Liebhold, A. , W. D. Koenig , and O. N. Bjørnstad . 2004. Spatial synchrony in population dynamics. Annual Review of Ecology Evolution and Systematics 35:467–490.

[ecy3486-bib-0032] Loreau, M. 2010. From populations to ecosystems: Theoretical foundations for a new ecological synthesis (MPB‐46). Volume 50. Princeton University Press, Princeton, New Jersey, USA.

[ecy3486-bib-0033] Loreau, M. , and C. de Mazancourt . 2008. Species synchrony and its drivers: neutral and nonneutral community dynamics in fluctuating environments. American Naturalist 172:E48–E66. 10.1086/589746 18598188

[ecy3486-bib-0034] Loreau, M. , and C. de Mazancourt . 2013. Biodiversity and ecosystem stability: a synthesis of underlying mechanisms. Ecology Letters 16:106–115. 23346947 10.1111/ele.12073

[ecy3486-bib-0035] Micheli, F. , K. l. Cottingham , J. Bascompte , O. n. Bjornstad , G. l. Eckert , J. M. Fischer , T. H. Keitt , B. E. Kendall , J. l. Klug , and J. A. Rusak . 1999. The dual nature of community variability. Oikos 85:161–169.

[ecy3486-bib-0036] Moran, P. A. P. 1953. The statistical analysis of the Canadian Lynx cycle II. Synchronization and meteorology. Australian Journal of Ecology 1:291–298.

[ecy3486-bib-0037] Myers, J. A. , J. M. Chase , R. M. Crandall , and I. Jiménez . 2015. Disturbance alters beta‐diversity but not the relative importance of community assembly mechanisms. Journal of Ecology 103:1291–1299.

[ecy3486-bib-0038] Peco, B. , T. Espigares , and C. Levassor . 1998. Trends and uctuations in species abundance and richness in Mediterranean annual pastures. Applied Vegetation Science 1:21–28.

[ecy3486-bib-0039] Pitt, M. , and H. Heady . 1978. Responses of annual vegetation to temperature and rainfall patterns in northern California. Ecology 59:336–350.

[ecy3486-bib-0040] Ripa, J. , and P. Lundberg . 1996. Noise colour and the risk of population extinctions. Proceedings of the Royal Society B 263:1751–1753.

[ecy3486-bib-0041] Rypel, A. L. , and S. R. David . 2017. Pattern and scale in latidude‐production relationships for freshwater fishes. Ecosphere 8:e01660.

[ecy3486-bib-0042] Schindler, D. E. , J. B. Armstrong , and T. E. Reed . 2015. The portfolio concept in ecology and evolution. Frontiers in Ecology and the Environment 13:257–263.

[ecy3486-bib-0043] Schreiber, T. , and A. Schmitz . 2000. Surrogate time series. Physica D: Nonlinear Phenomena 142:346–382.

[ecy3486-bib-0044] Schulze, E.‐D. , and H. A. Mooney . 2012. Biodiversity and ecosystem function. Springer Science and Business Media, Heidelberg, Germany.

[ecy3486-bib-0045] Shoemaker, L. G. , and B. A. Melbourne . 2016. Linking metacommunity paradigms to spatial coexistence mechanisms. Ecology 97:2436–2446. 27859071 10.1002/ecy.1454

[ecy3486-bib-0046] Tilman, D. , and J. A. Downing . 1994. Biodiversity and stability in grasslands. Nature 367:363–365.

[ecy3486-bib-0047] Walter, J. A. , L. M. Hallett , L. W. Sheppard , T. L. Anderson , L. Zhao , R. J. Hobbs , K. N. Suding , and D. C. Reuman . 2021. Micro‐scale geography of synchrony in a serpentine plant community. Journal of Ecology 109:750–762.

[ecy3486-bib-0048] Walter, J. A. , L. W. Sheppard , T. L. Anderson , J. H. Kastens , O. N. Bjørnstad , A. M. Liebhold , and D. C. Reuman . 2017. The geography of spatial synchrony. Ecology Letters 20:801–814. 28547786 10.1111/ele.12782

[ecy3486-bib-0049] Wang, S. , T. Lamy , L. M. Hallett , and M. Loreau . 2019. Stability and synchrony across ecological hierarchies in heterogeneous metacommunities: linking theory to data. Ecography 42:1200–1211.

[ecy3486-bib-0050] Wang, S. , and M. Loreau . 2014. Ecosystem stability in space: alpha, beta, and gamma variability. Ecology Letters 17:891–901. 24811401 10.1111/ele.12292

[ecy3486-bib-0051] Wilcox, K. R. , et al. 2017. Asynchrony among local communities stabilizes ecosystem function of metacommunities. Ecology Letters 20:1534–1545. 29067791 10.1111/ele.12861 PMC6849522

[ecy3486-bib-0052] Worm, B. , and J. E. Duffy . 2003. Biodiversity, productivity and stability in real food webs. Trends in Ecology & Evolution 18:628–632.

