# Supplementary material for: The spatial synchrony of species richness and its relationship to ecosystem stability
Source: Ecology. 2021 Aug 12;102(11):e03486. doi: 10.1002/ecy.3486 (PMC9286696; doi:10.1002/ecy.3486)
Supplement: Supplementary file 1 — Appendix S1 [file ECY-102-0-s001.pdf]

**Supporting Information.** Walter, J.A., L.G. Shoemaker, N.K. Lany, M.C.N. Castorani, S.B. Fey, J.C. Dudney, C. Portales-Reyes, A.L. Rypel, K.L. Cottingham, K.N. Suding, D.C. Reuman, and L.M. Hallett. 2021. The spatial synchrony of species richness and its relationship to ecosystem stability. Ecology.

**Appendix S1**

# S1 Dataset descriptions

Data comprise the abundance (measured as percent cover or biomass) of primary producer taxa measured on fixed area, permanent quadrats. Each dataset was prepared by retaining only plots that were sampled in all years and were not part of an experimental treatment, removing entries for unknown taxa, and separating into separate datasets for different communities if applicable. In cases where quadrats were not permanently marked, but rather were laid out randomly each sampling interval, abundances within each quadrat were aggregated to level of the unit of observation over time (typically the transect level). Taxa were identified to the genus or species level. We removed very rare taxa that did not occur in at least 5% of all plot-year combinations. We visually evaluated temporal changes in richness as well as species accumulation curves in both time and space.

## S1.1 Florida Keys (DRT, LOK, MDK, UPK)

Data on the percent cover of corals in in Florida (Dry Tortugas National Park [DRT], Lower Florida Keys [LOK], Middle Florida Keys [MDK], and Upper Florida Keys [UPK]) were downloaded from the U.S. Geological Survey (Guest et al., 2018). At each of 40 sites, coral cover was estimated annually (1996-2015) within 2-4 permanent transects per site (40 cm wide by 22 m long), with each transect separated by 1 m. Each site represented one of three possible habitats (deep forereef,

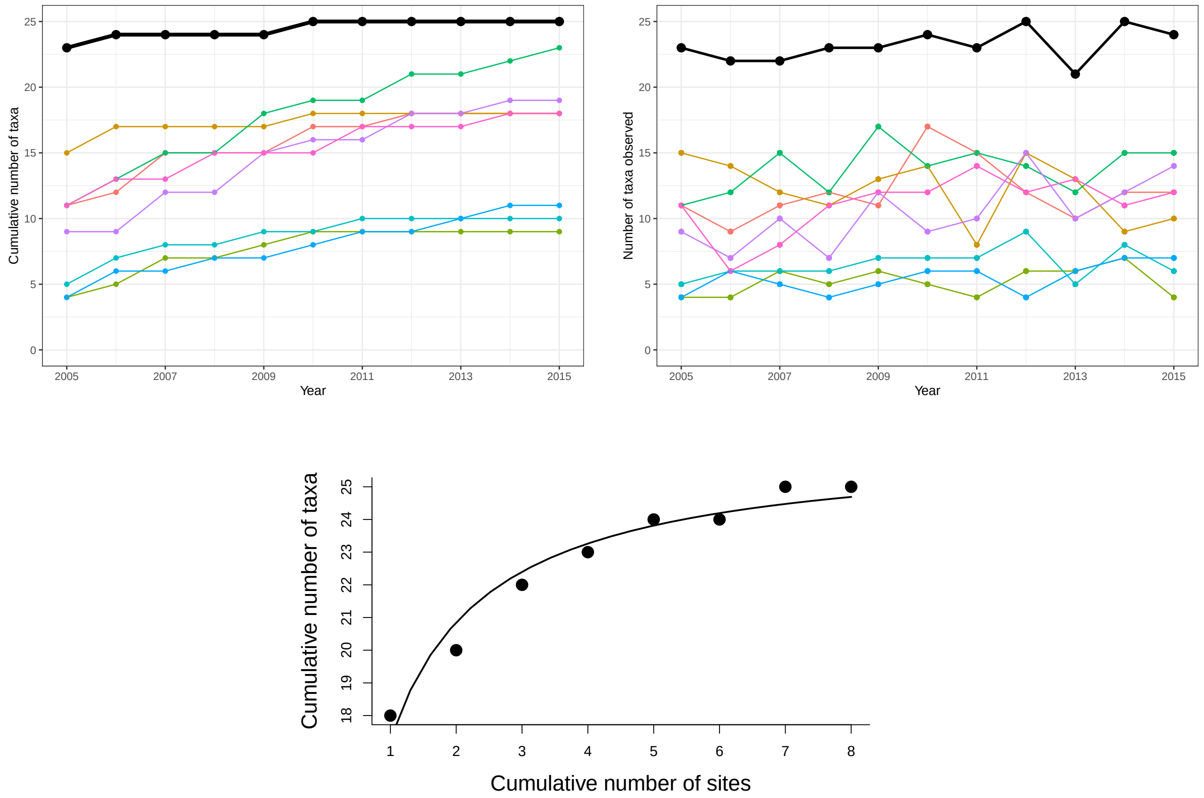


Figure S1: Temporal species accumulation curves (upper left), annual richness (upper right), and spatial species accumulation curve (lower) for 6 Dry Tortugas, Florida Keys corals plots (2005-2015). The black lines represent total site-level values across all plots.

shallow forereef, and patch reef). Cover was aggregated to the site scale. Data are shown in Figures S1-S4.

## S1.2 Hayes, Kansas (HAY)

Data on plant percent cover were obtained for 13 $1m^2$ quadrats in mixed grass prairie habitat at Hay, Kansas over the period 1943-1972 (Adler et al., 2007). Taxa were identified to the species level. Data are shown in Figure S5.

## S1.3 Jasper Ridge (JRG)

Jasper Ridge Biological Preserve is a serpentine grassland. Data consist of percent cover for 25 grasses and forbs identified to the species level from a long-term experiment begun in 1983 (Hobbs

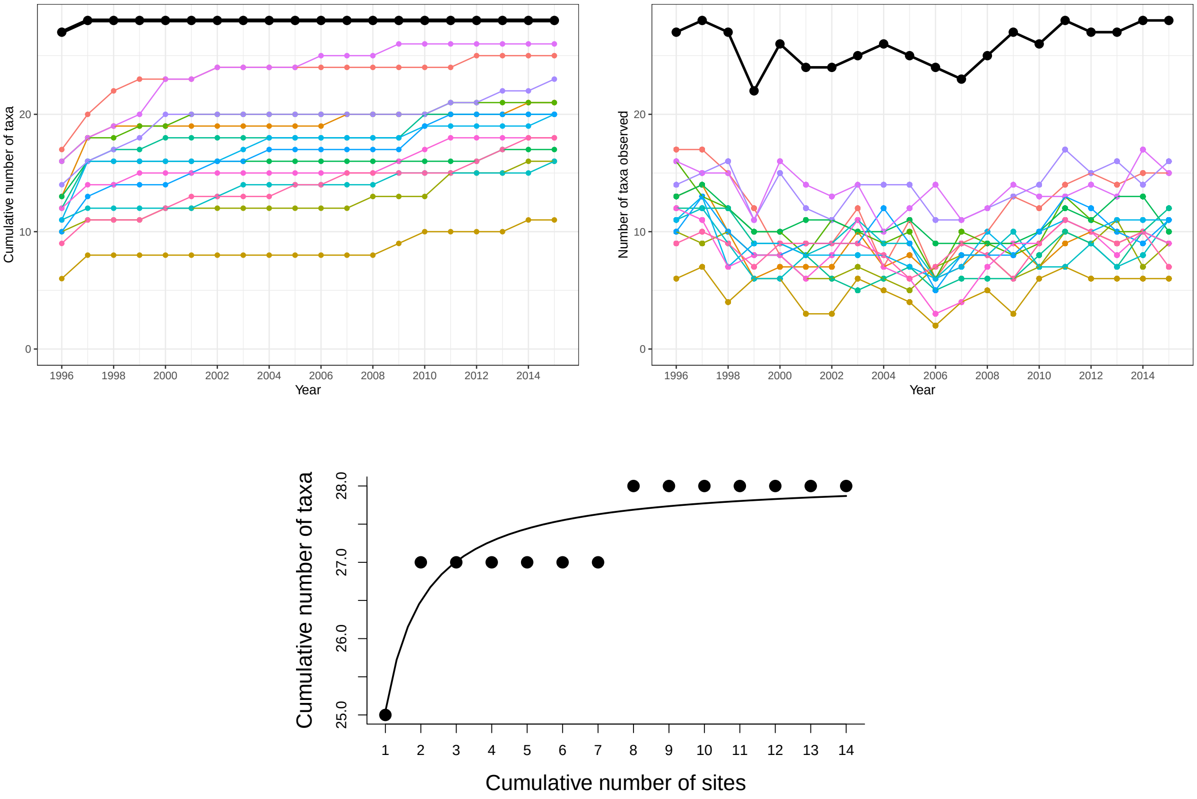


Figure S2: Temporal species accumulation curves (upper left), annual richness (upper right), and spatial species accumulation curve (lower) for 14 Lower Florida Keys corals plots (1996-2015). The black lines represent total site-level values across all plots.

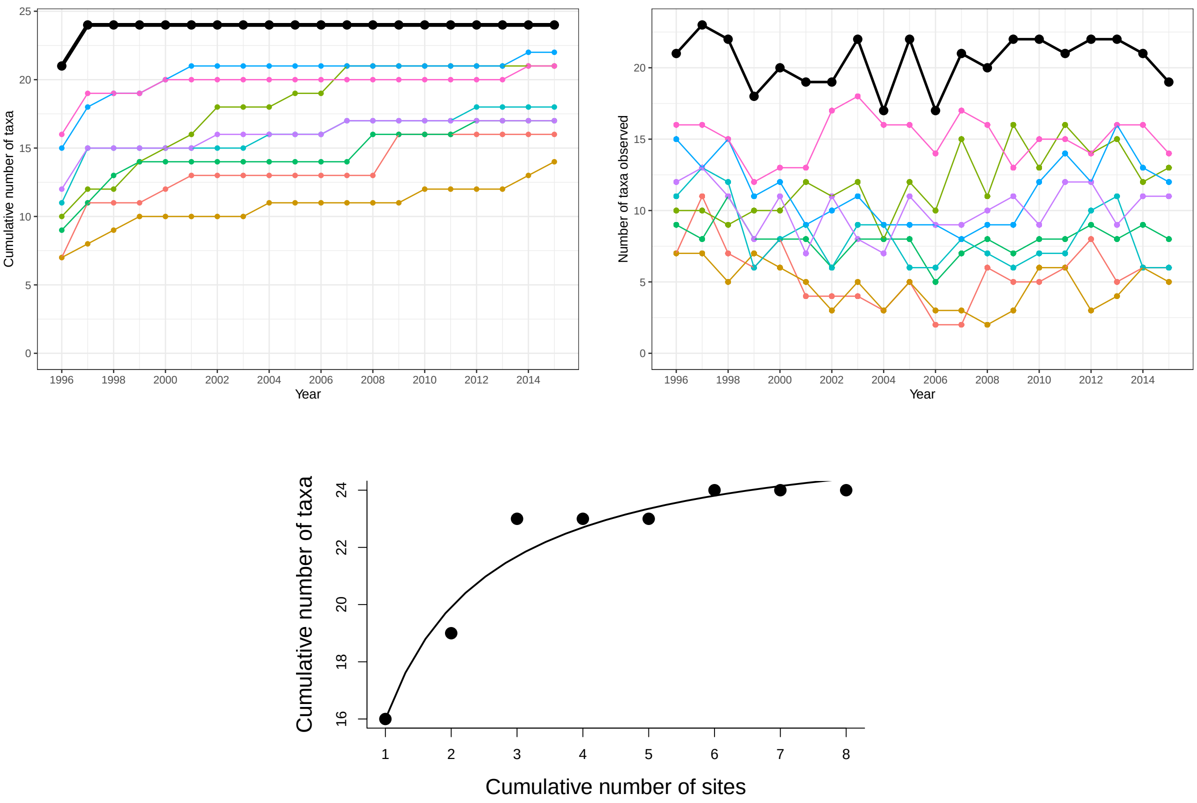


Figure S3: Temporal species accumulation curves (upper left), annual richness (upper right), and spatial species accumulation curve (lower) for 8 Middle Florida Keys corals plots (1996-2015). The black lines represent total site-level values across all plots.

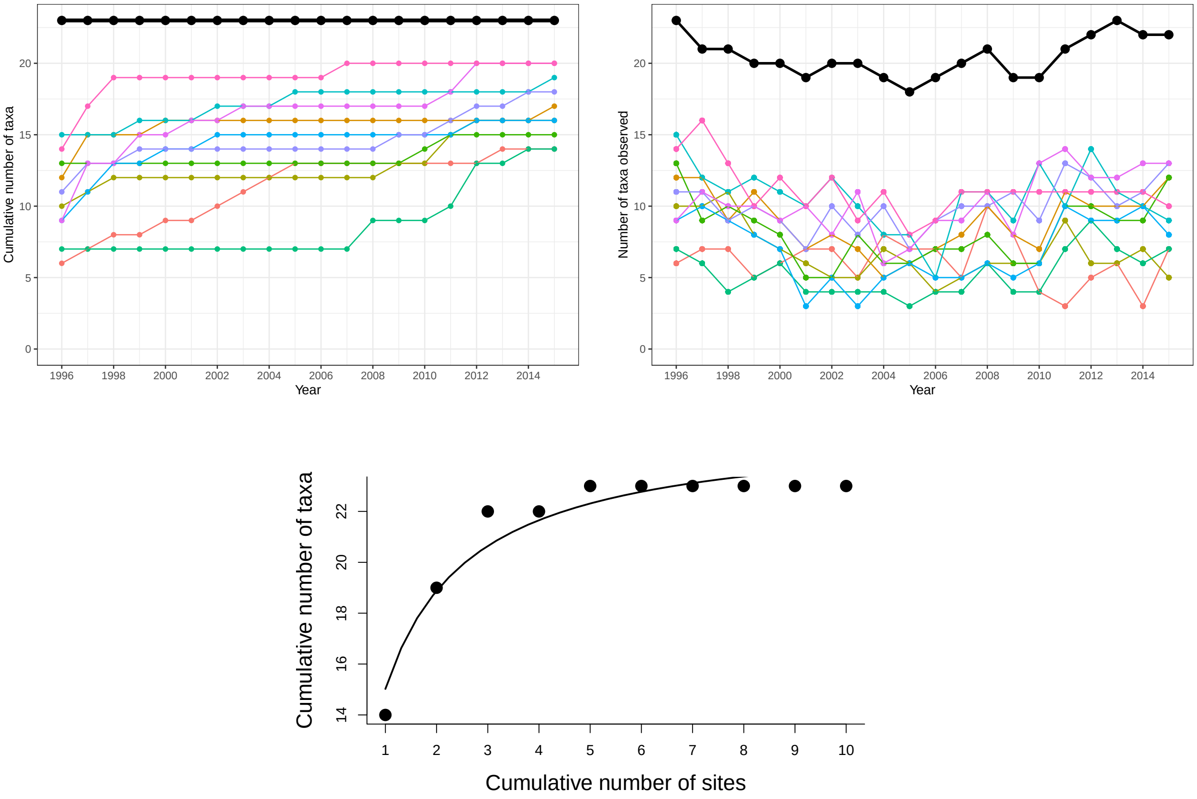


Figure S4: Temporal species accumulation curves (upper left), annual richness (upper right), and spatial species accumulation curve (lower) for 10 Upper Florida Keys corals plots (1996-2015). The black lines represent total site-level values across all plots.

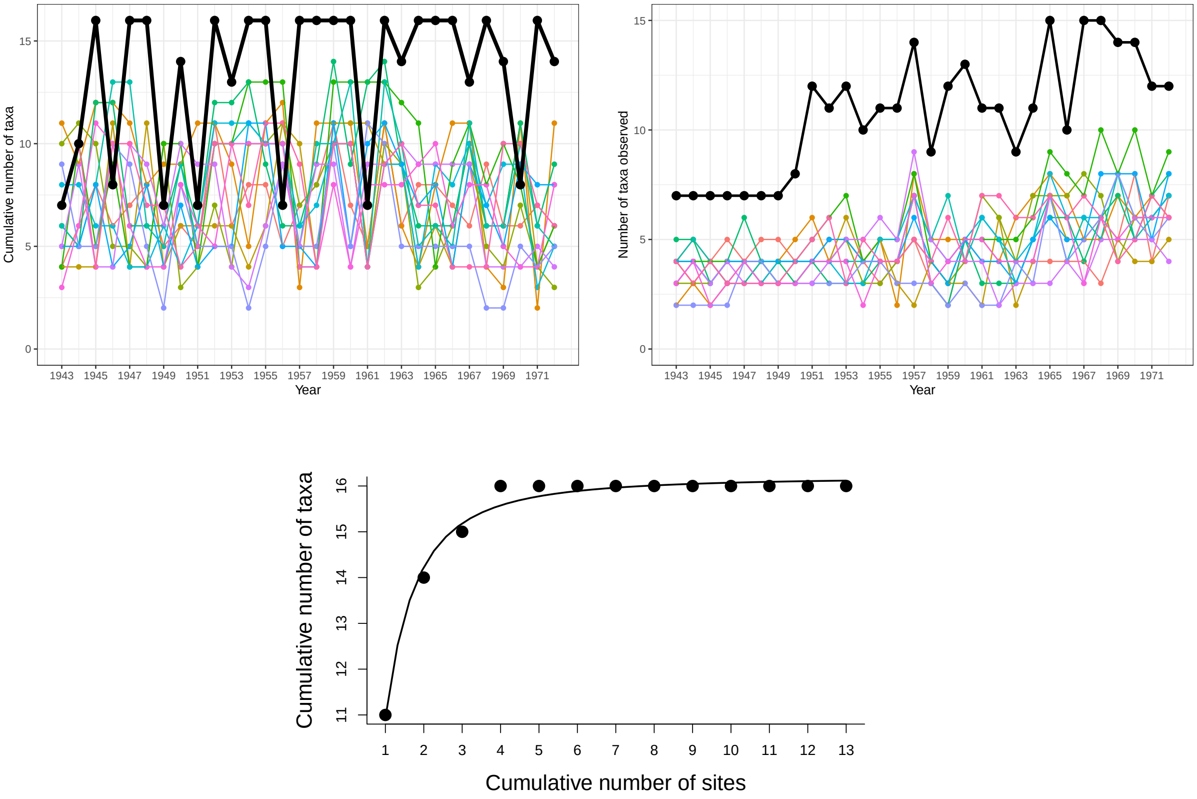


Figure S5: Temporal species accumulation curves (upper left), annual richness (upper right), and spatial species accumulation curve (lower) for 13 plots at Hay, Kansas (1943-1972). The black lines represent total site-level values across all plots.

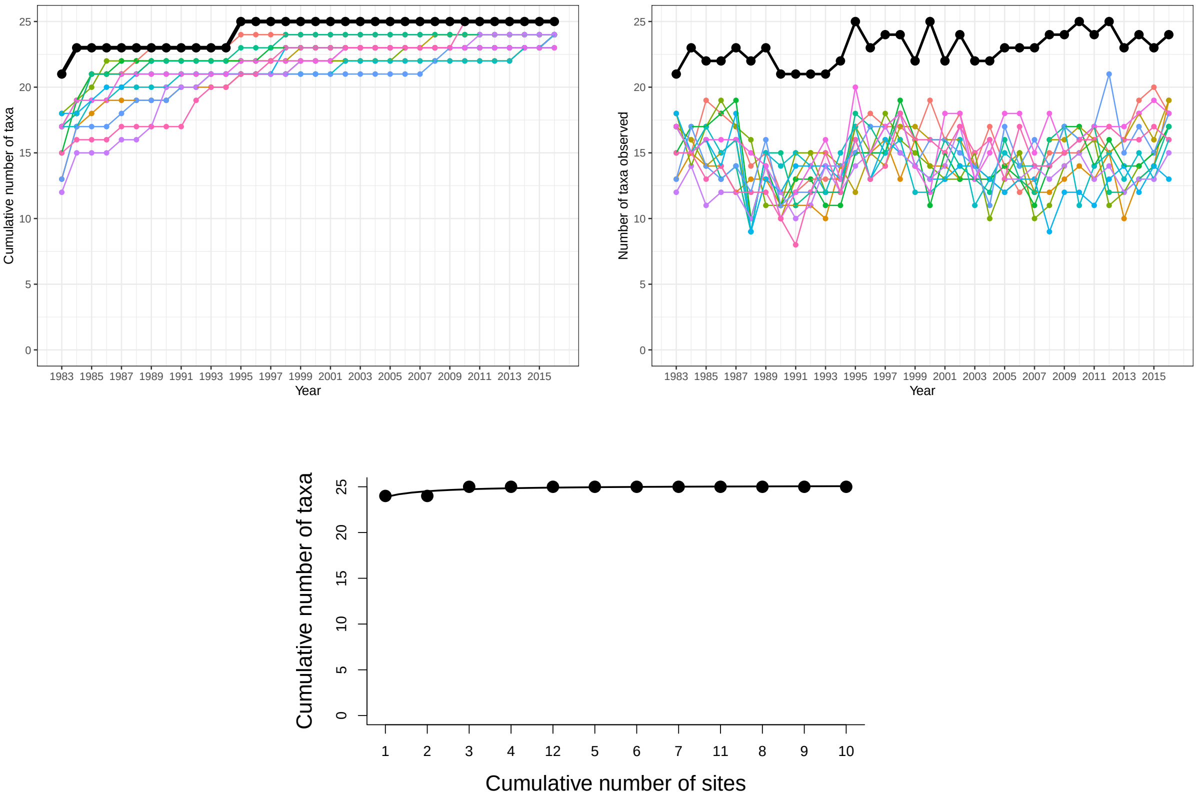


Figure S6: Temporal species accumulation curves (left), annual richness (right), and spatial species accumulation curve (lower) for 12 plots at Jasper Ridge, CA (1983-2016). The black lines represent total site-level values across all plots.

and Mooney, 1985) and sampled continuously on an annual time interval. The experiment consists of control, gopher exclosure, and rabbit exclosure plots in a nested block design: there are three replicates of each treatment and four $1m^2$ plots within each replicate. Data were obtained from Lauren Hallett on May 11, 2017. We analyzed plots in the control treatment only and considered the $1m^2$ plots the spatial unit of interest, for a total of 12 plots from 1983 to 2016 (Figure S6).

## S1.4 Jornada (JRN)

Data on the biomass of plant species at the Jornada LTER through 2014 were obtained from Peters and Huenneke (2015). The grassland habitat was chosen at Jornada to be consistent across LTER site comparisons and exclude primarily shrub-dominated ecosystems. Within the grassland, 3 projects were included: BASN, IBPE, and SUMM, which are denoted as three distinct communities

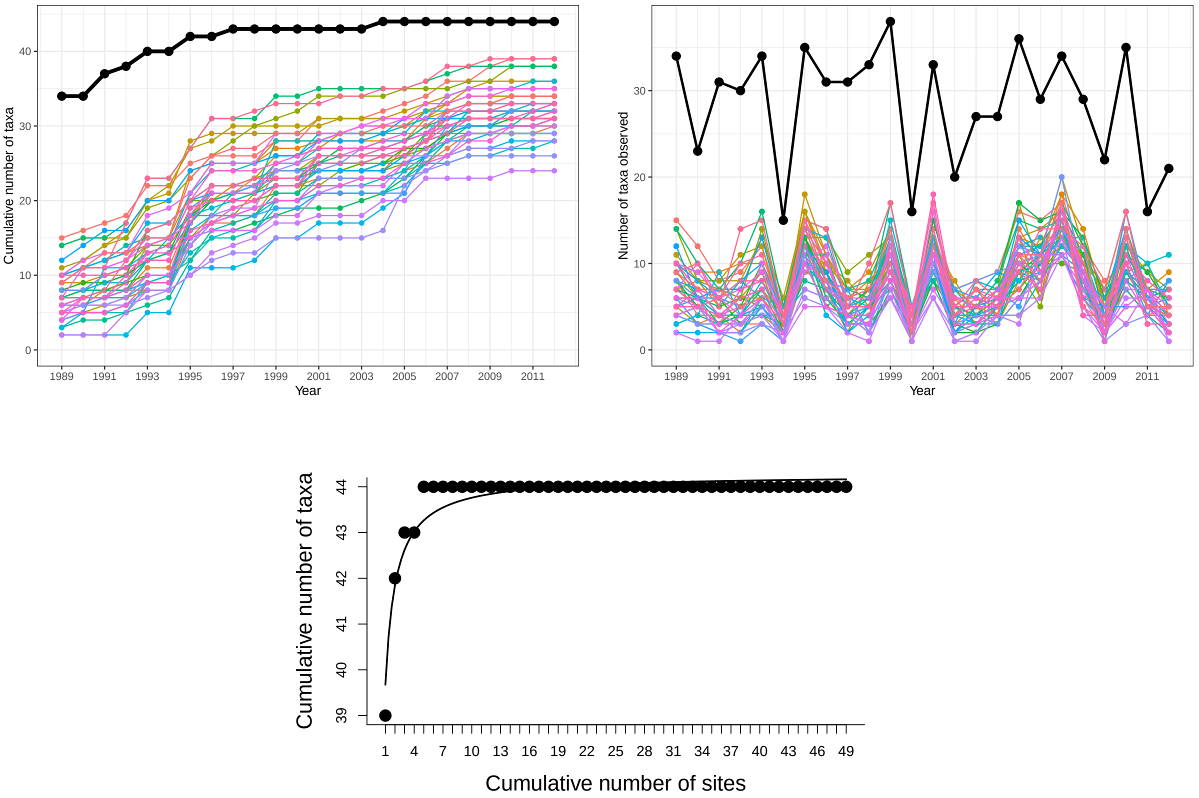


Figure S7: Temporal species accumulation curves (left), annual richness (right), and spatial species accumulation curve (lower) for 49 plots comprising the Basin (BASN) grassland community at Jornada LTER (1989-2012). The black lines represent total site-level values across all plots.

and analyzed separately. All 49 plots within each habitat type were included in our analyses and all sampling intervals between 1989 and 2012 (Figures S7, S8, and S9).

## S1.5 Konza (KNZ)

Data on percent canopy cover were recorded in two different soil types in in watershed 001d (Hartnett and Collins, 2016) We analyzed the data from the the fertile, nonrocky tully (lowland) and shallow, rocky florence (upland) soil types separately. The spatial and temporal species accumulation curves level off, indicating that the community is well sampled and new species are not immigrating into the community (Figures S10 and S11).

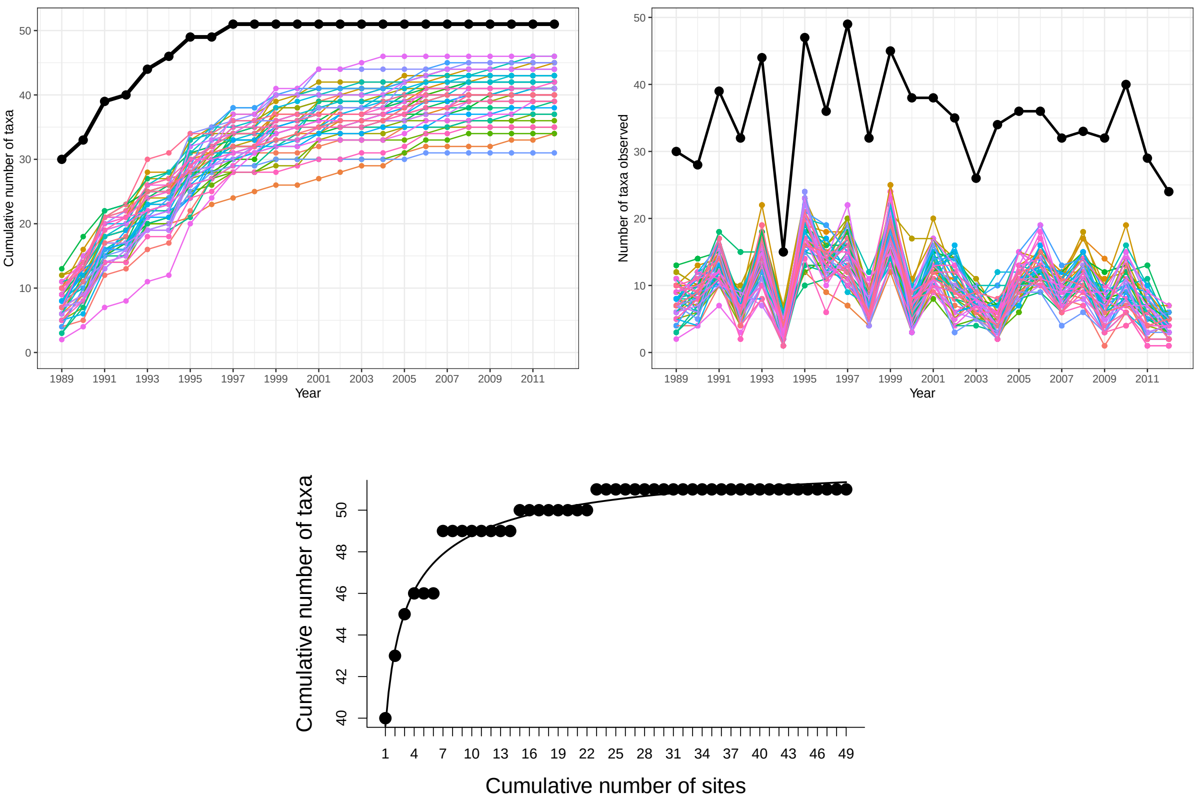


Figure S8: Temporal species accumulation curves (left), annual richness (right), and spatial species accumulation curve (lower) for 49 plots comprising the IBPE grassland community at Jornada LTER (1989-2012). The black lines represent total site-level values across all plots.

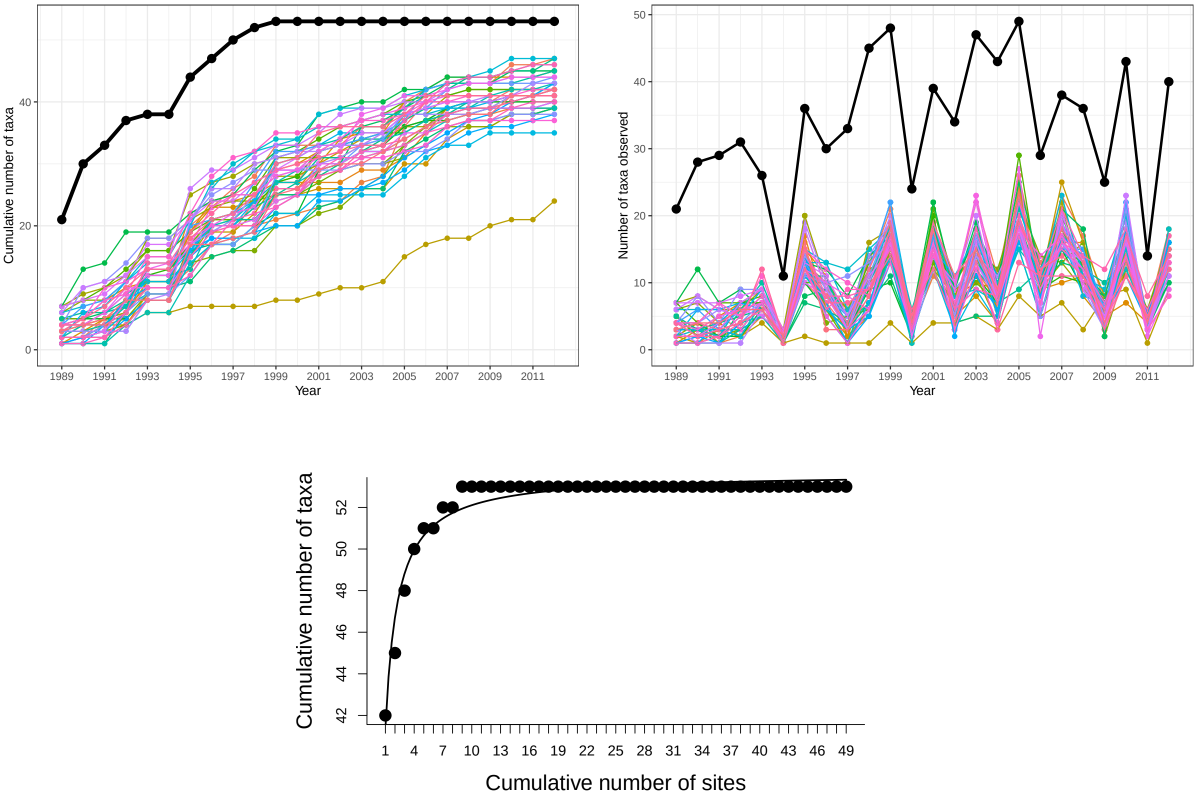


Figure S9: Temporal species accumulation curves (left), annual richness (right), and spatial species accumulation curve (lower) for 49 plots comprising the Summerford Mountain (SUMM) grassland community at Jornada LTER (1989-2012). The black lines represent total site-level values across all plots.

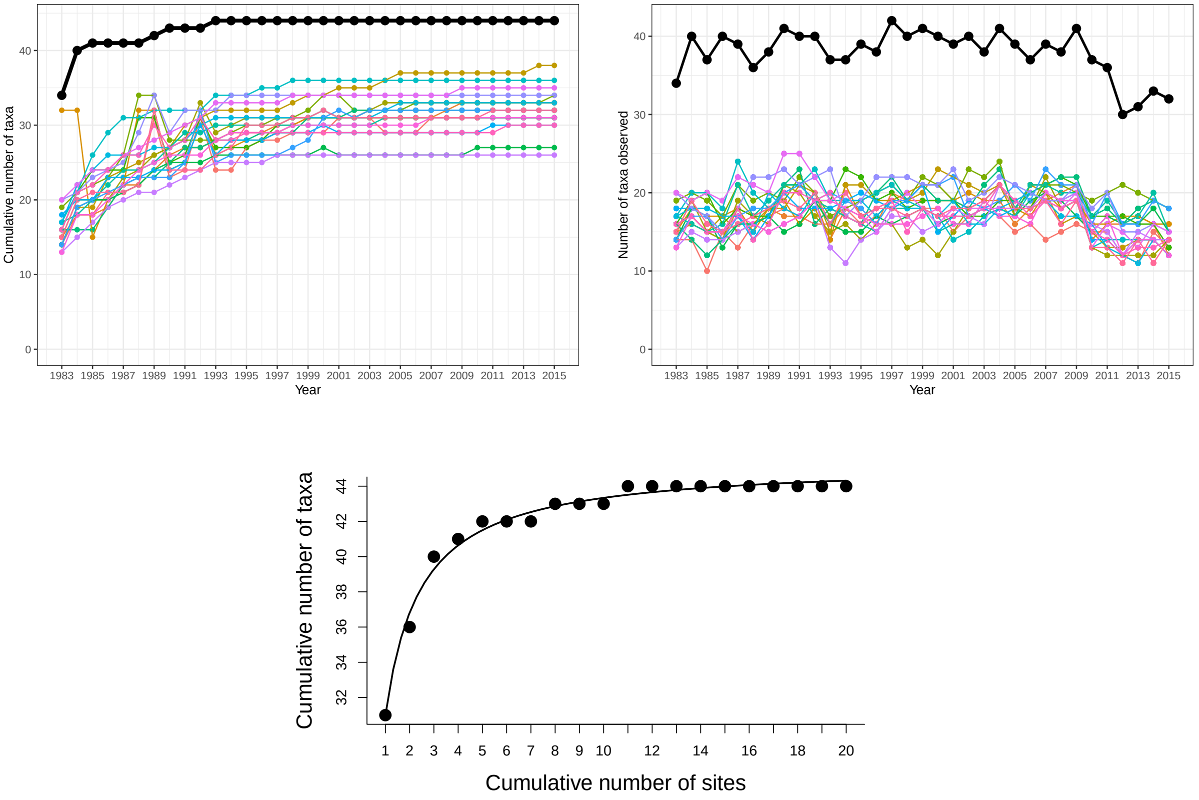


Figure S10: Temporal species accumulation curves (left), annual richness (right), and spatial species accumulation curve (lower) for 40 plots comprising fertile, nonrocky tully (lowland) soil habitats at Konza LTER (1983-2015). The black lines represent total site-level values across all plots.

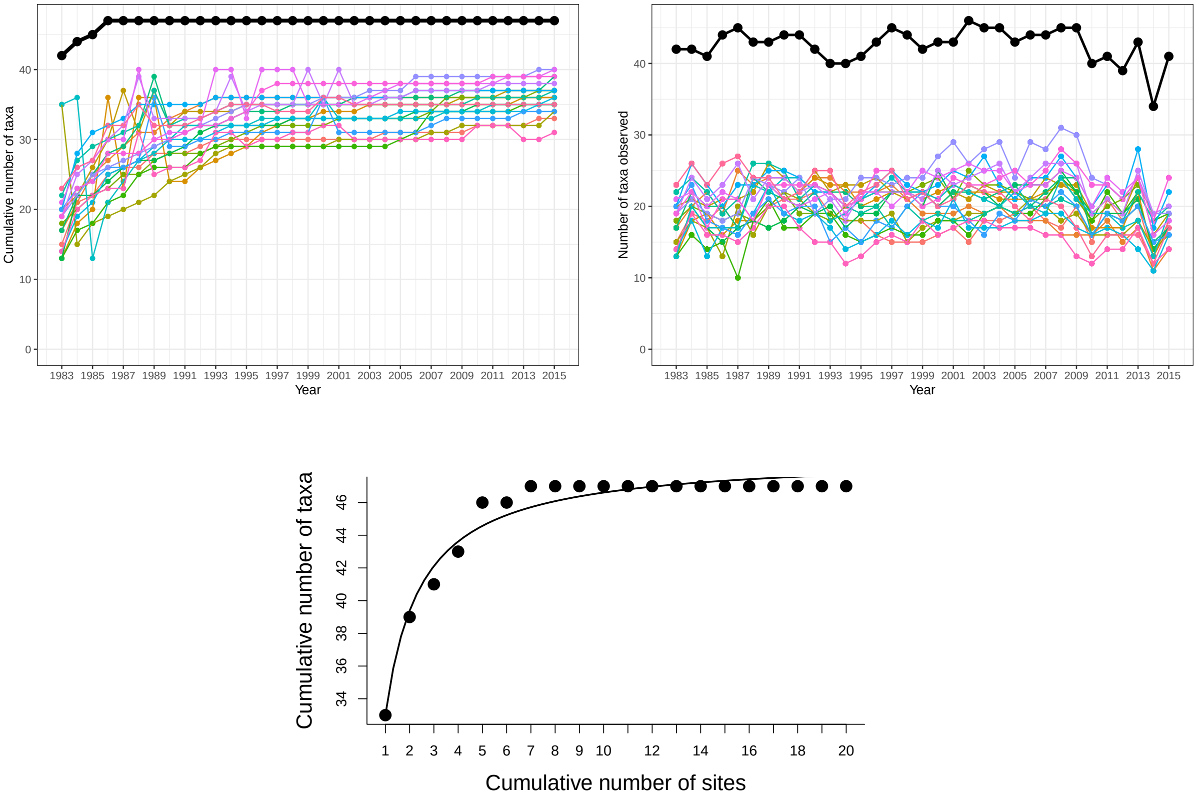


Figure S11: Temporal species accumulation curves (left), annual richness (right), and spatial species accumulation curve (lower) for 40 plots comprising the shallow, rocky florence (upland) soil habitat at Konza LTER (1983-2015). The black lines represent total site-level values across all plots.

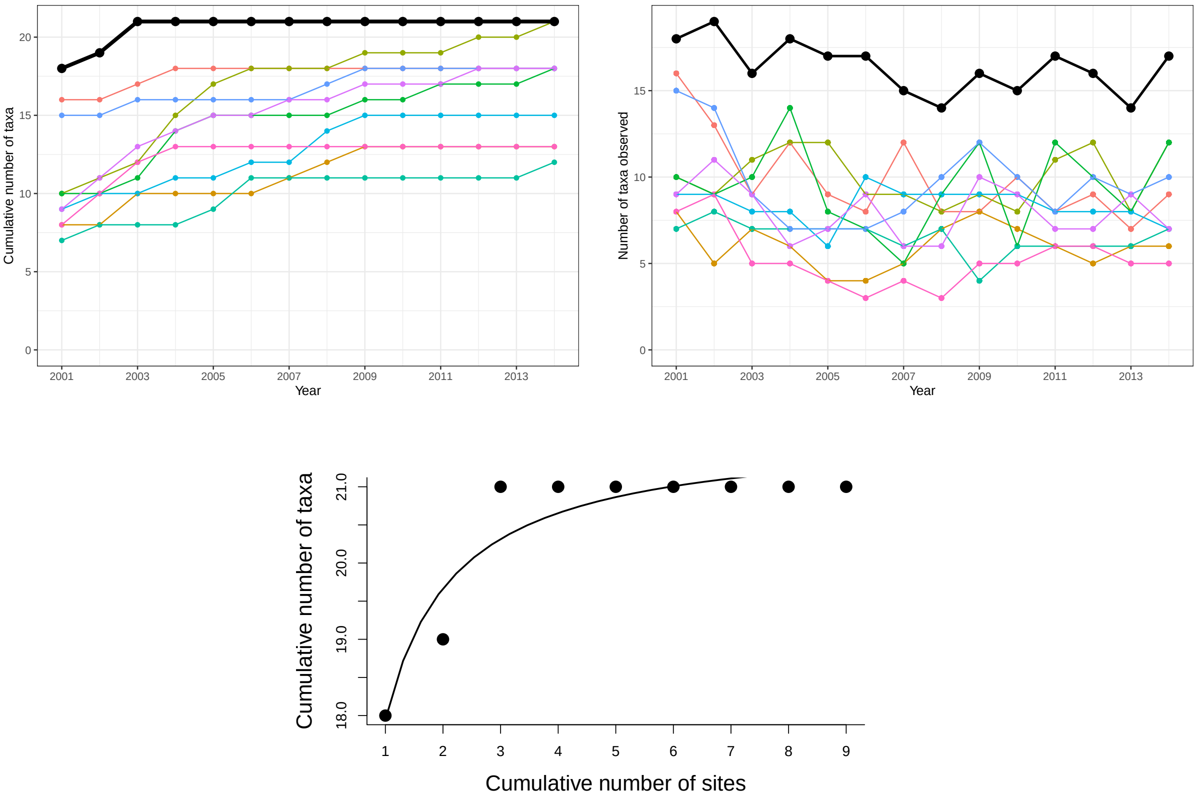


Figure S12: Temporal species accumulation curves (upper left), annual richness (upper right), and spatial species accumulation curve (lower) for 9 Maui, Hawaii corals plots (1999-2015). The black lines represent total site-level values across all plots.

## S1.6 Maui, Hawaii (MAU)

Data on the percent cover of corals in Maui, Hawaii were downloaded from the U.S. Geological Survey (Guest et al., 2018). At each of 9 sites, coral cover was estimated annually (1999-2014) within ten 10-m transects using 20 photoquadrats per transect (bottom area = 0.34 $m^2$). Photoquatrats were not permanent, and cover was aggregated to the site scale. Data are shown in Figure S12.

## S1.7 Moorea Coral Reef (MCR)

Data on the percent cover of coral and algae taxa in three different reef habitats the Moorea Coral Reef LTER were downloaded from EDI (Moorea Coral Reef LTER and Carpenter, 2015; Moorea Coral Reef LTER and Edmunds, 2018). At each of 30 sites, coral cover was estimated annually (2006-2015) within one permanent 40-m transect at each site using 40 photoquadrats per transect

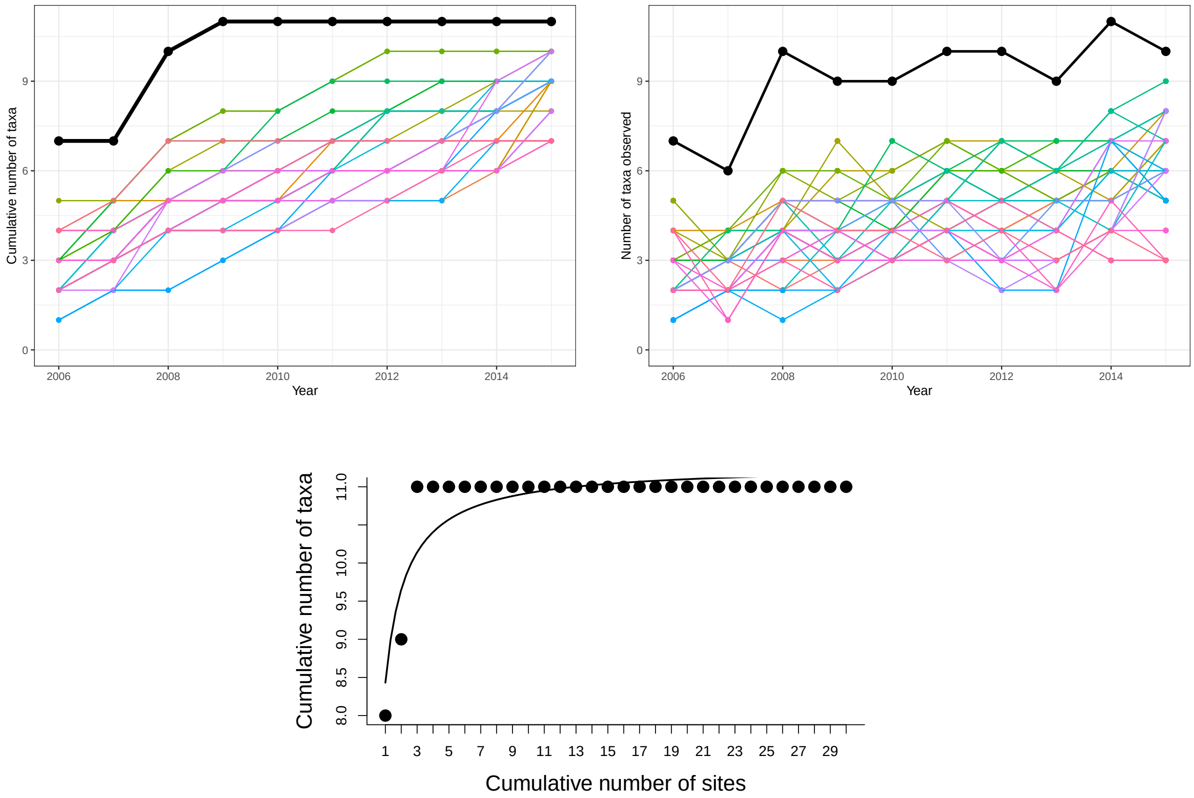


Figure S13: Temporal species accumulation curves (left), annual richness (right), and spatial species accumulation curve (lower) for the backreef habitat at Moorea Coral Reef (2006-2015). The black lines represent total site-level values across all plots.

(bottom area = 0.25 $m^2$). Each site represented one of three possible habitats (back reef, fringing reef, and outer reef). The data are shown from the back, fringing, and outer reef habitats in Figures S13, S14, and S15, respectively.

## S1.8 Santa Barbara Coastal (SBC)

Annual estimates of biomass of all macroalgal taxa in kelp forests in the Santa Barbara Coastal LTER were downloaded from EDI (Santa Barbara Coastal LTER and Reed, 2018). At each of 11 sites, macroalgal density or cover was surveyed within 2-8 permanent transects per site (2 m wide by 40 m long) (Harrer et al., 2013; Reed et al., 2016). Abundance and size were converted to dry biomass using taxon-specific relationships developed for the study region (Harrer et al., 2013; Reed et al., 2016; Rassweiler et al., 2018). Data are shown in Figure S16.

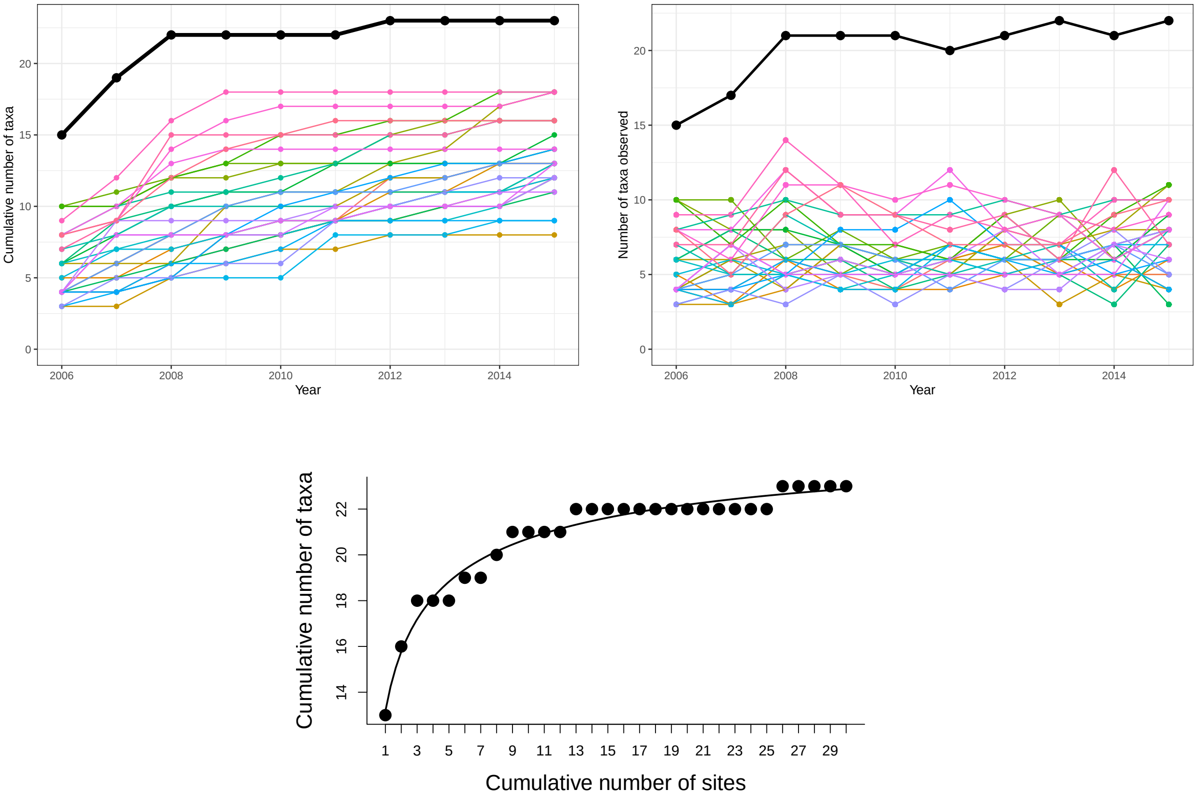


Figure S14: Temporal species accumulation curves (left), annual richness (right), and spatial species accumulation curve (lower) for algal and coral taxa in the fringing habitat at Moorea Coral Reef (2006-2015). The black lines represent total site-level values across all plots.

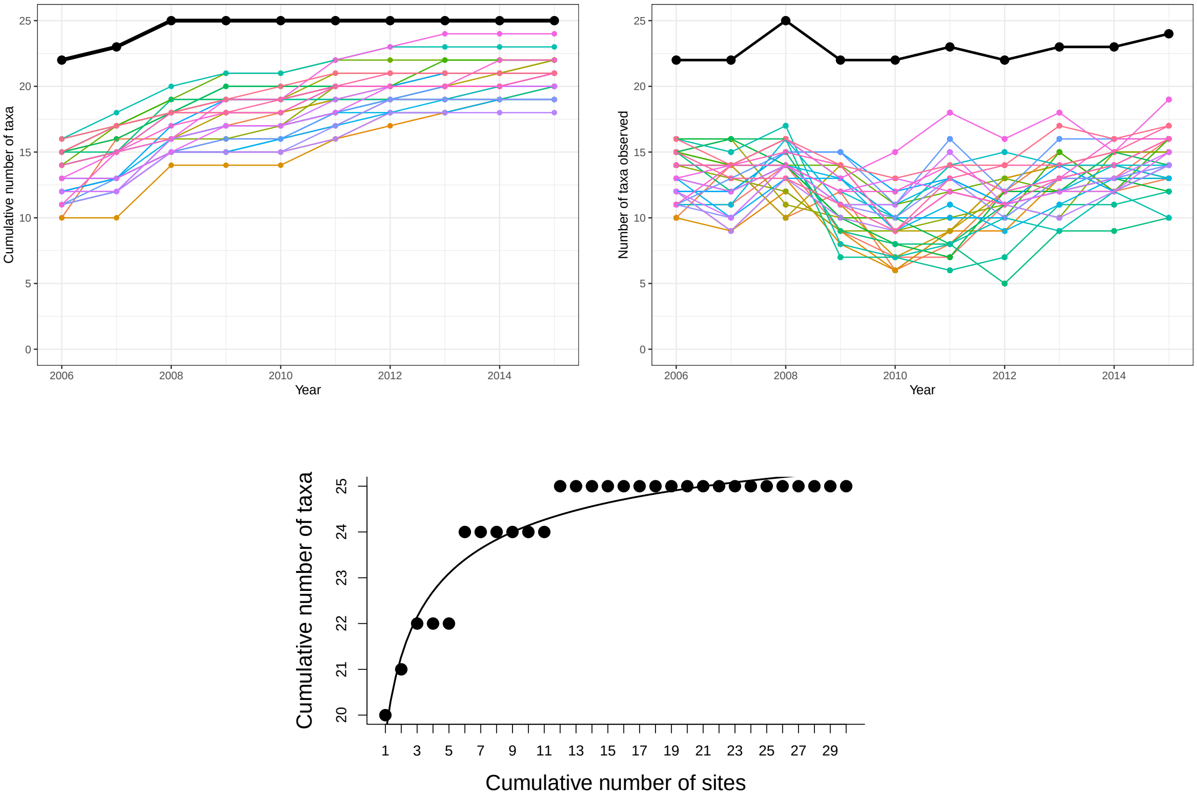


Figure S15: Temporal species accumulation curves (left), annual richness (right), and spatial species accumulation curve (lower) for algal and coral taxa in the outer (10 m depth) habitat at Moorea Coral Reef (2006-2015). The black lines represent total site-level values across all plots.

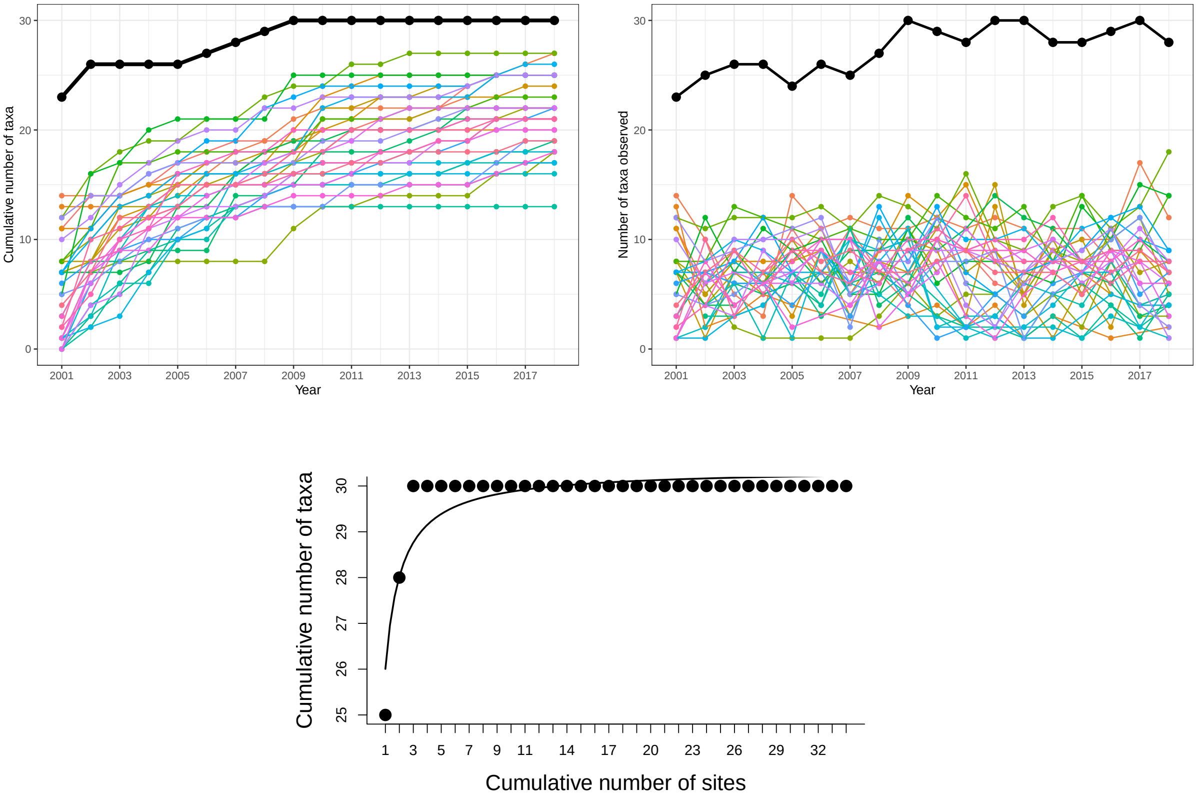


Figure S16: Temporal species accumulation curves (left), annual richness (right), and spatial species accumulation curve (lower) for sessile invertebrate and algal taxa at 34 plots at Santa Barbara Coastal LTER. The black lines represent site-level values.

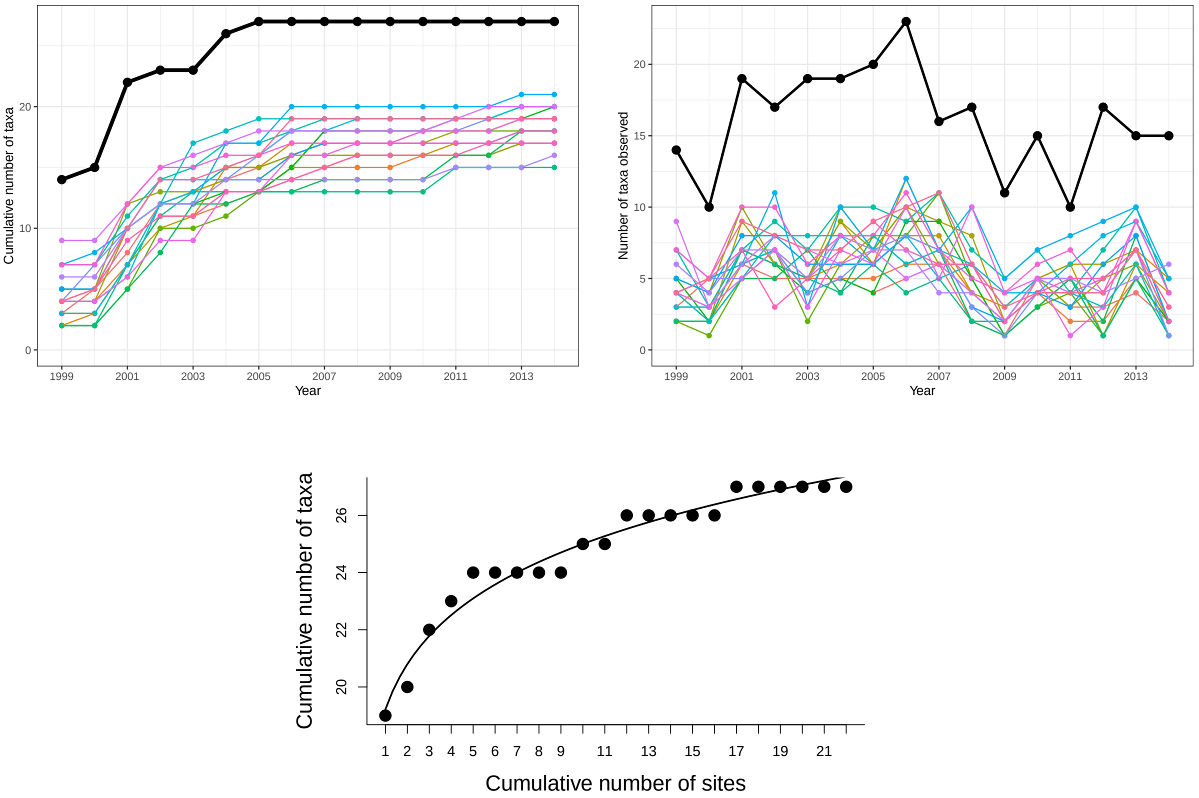


Figure S17: Temporal species accumulation curves (left) and annual richness (right), and spatial species accumulation curve (lower) in the black grama habitat at Sevilleta LTER. The black lines represent total site-level values across all plots.

## S1.9 Sevilleta (SEV)

Data were downloaded from EDI (Muldavin and Moore, 2016). Three ecosystem types were included: black gamma (G), creosote(C) and blue gramma (B) (Figures S17, S18, and S19). Methods are described in (Muldavin et al., 2008) and (Rudgers et al., 2018). Within the black gramma community (G), the 22 plots that were sampled annually between 1999-2014 were included. Within the creosote community (C), the plots that were sampled annually between 1999-2014 were included. The G and C communities are about 0.5 km apart. Within the blue gramma community (B), 42 taxa at 30 plots sampled annually between 2002-2014 were included.

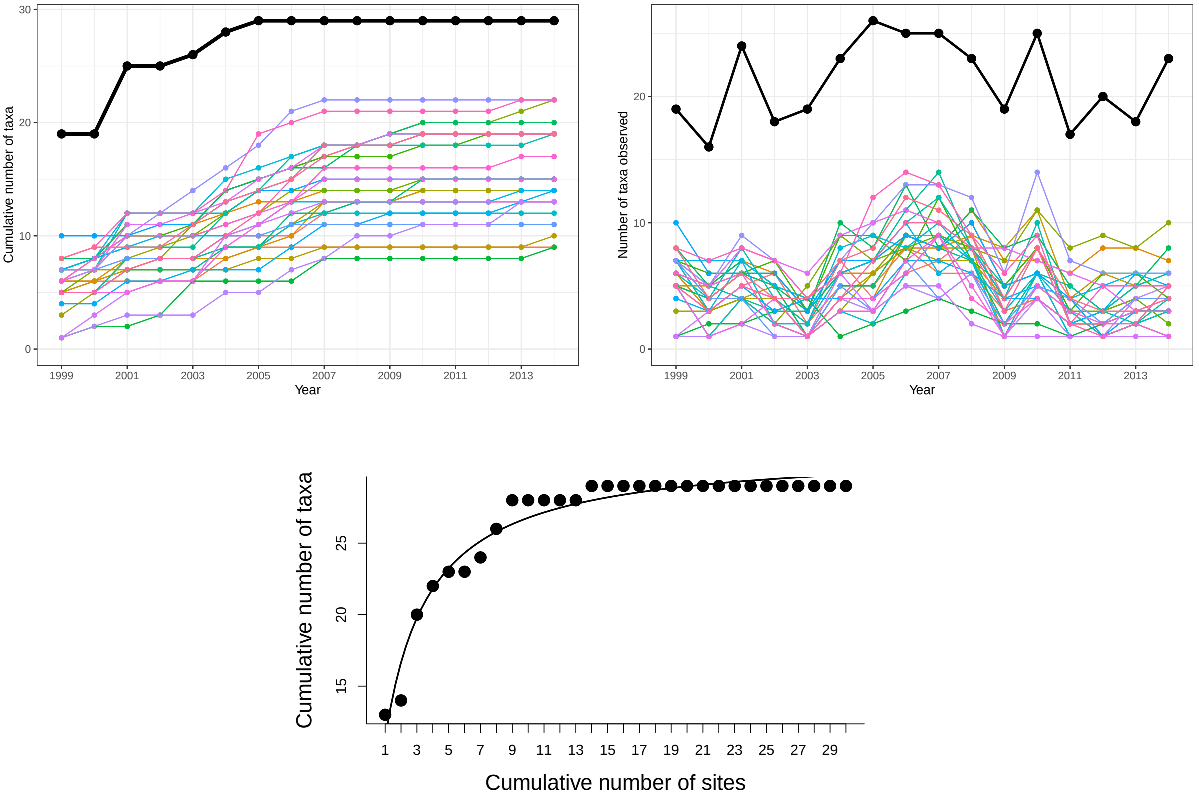


Figure S18: Temporal species accumulation curves (left) and annual richness (right), and spatial species accumulation curve (lower) in the creosote habitat at Sevilleta LTER. The black lines represent total site-level values across all plots.

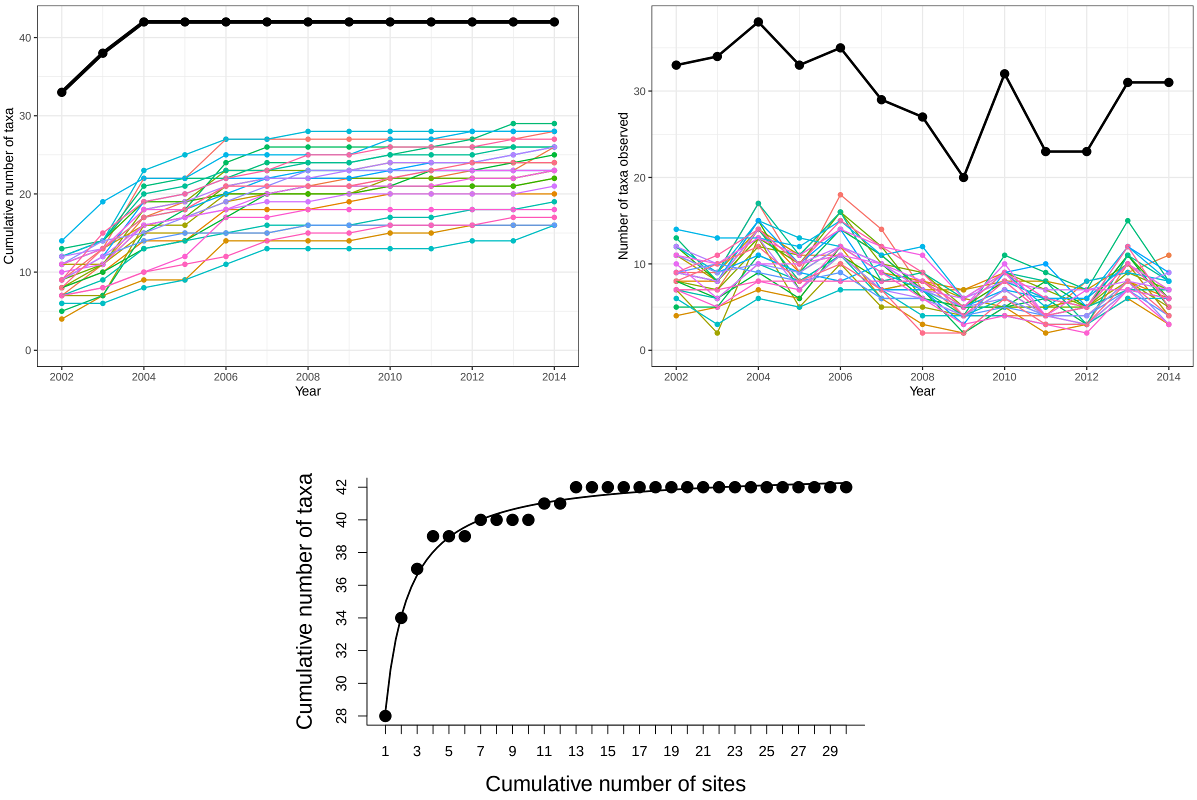


Figure S19: Temporal species accumulation curves (left) and annual richness (right), and spatial species accumulation curve (lower) in the blue grama habitat at Sevilleta LTER. The black lines represent total site-level values across all plots.

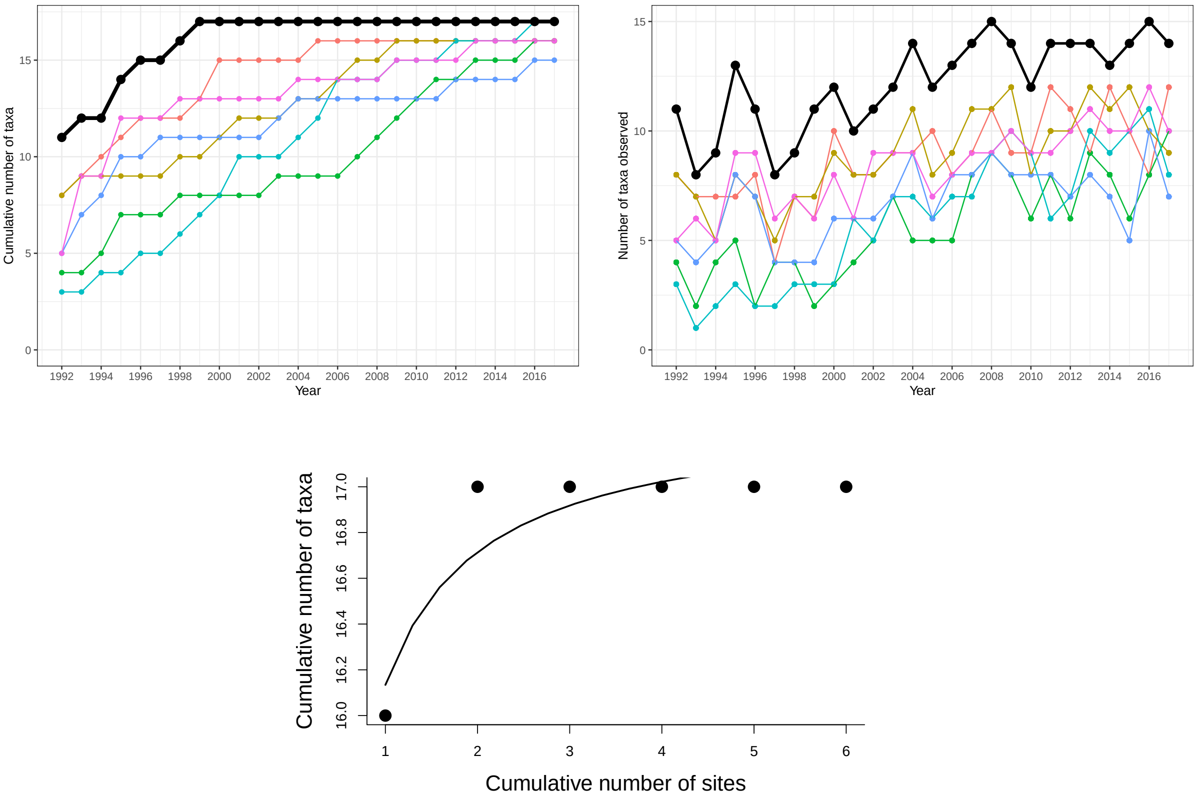


Figure S20: Temporal species accumulation curves (left), annual richness (right), and spatial species accumulation curve (lower) for coral taxa at CSUN US Virgin Islands research site. The black lines represent site-level values.

### S1.10 US Virgin Islands National Park (USVI

Data on the percent cover of Scleractinian corals at St. John in the US Virgin Islands were downloaded from EDI (Edmunds, 2019). At each of 6 sites, Coral cover was estimated using 18-40 photoquadrates (bottom area = 0.25 $m^2$). Corals were identified to the genus level. Quadrats were not permanent, and abundance was aggregated to the site scale. Data are shown in Figure S20.

## S2 Statistical significance of richness synchrony

Table S1: The degree of spatial synchrony in species richness ($r$) and its associated $p$-value, for 20 empirical metacommunity datasets.

| Dataset | $r$ | $p$ |
|---:|---|---:|
| DRT | 0.054 | 0.177 |
| HAY | 0.396 | $< 0.001$ |
| JRG | 0.348 | $< 0.001$ |
| JRN_BASN | 0.760 | $< 0.001$ |
| JRN_IBPE | 0.775 | 0.001 |
| JRN_SUMM | 0.810 | $< 0.001$ |
| KNZ_UP | 0.389 | $< 0.0001$ |
| KNZ_LOW | 0.472 | $< 0.001$ |
| LOK | 0.519 | $< 0.001$ |
| MAU | 0.089 | 0.052 |
| MCR_BACK | 0.089 | $< 0.001$ |
| MCR_FRNG | 0.057 | 0.005 |
| MCR_OUT | 0.442 | $< 0.001$ |
| MDK | 0.236 | 0.001 |
| SBC | 0.053 | $< 0.001$ |
| SEV_B | 0.640 | $< 0.001$ |
| SEV_C | 0.611 | $< 0.001$ |
| SEV_G | 0.602 | $< 0.001$ |
| UPK | 0.392 | $< 0.001$ |
| USVI | 0.269 | $< 0.001$ |

## S3 Relationships between stability and community variables

Table S2: Linear regression relationships between stability and community variables, in simulated and empirical metacommunities. Stability is measured as the -1 × CV over time of total biomass or percent cover.

| | Simulated | | Empirical | |
|---|---|---|---|---|
| Predictor | $\beta$(SE) | $R^2$ | $\beta$(SE) | $R^2$ |
| Richness synchrony | -0.29(0.01) | 0.22 | -0.58(0.16) | 0.43 |
| Richness | 0.004(0.0003) | 0.08 | 0.019(0.011) | 0.13 |
| Evenness | 0.65(0.04) | 0.10 | 0.75(0.37) | 0.18 |
| $\beta$-diversity | 0.69(0.07) | 0.04 | 0.74(0.40) | 0.16 |
| Turnover rate | -3.79(0.32) | 0.05 | -1.03(0.25) | 0.49 |

## S4 Results from alternate model formulation

All following figures (Figures S21-S24) have identical formatting to those in the main text; the difference between these results and those presented in the main text is that, here, simulations used global dispersal but were otherwise identical. For results presented in the main text, dispersal was restricted to nearest neighbor patches. Results are largely consistent.

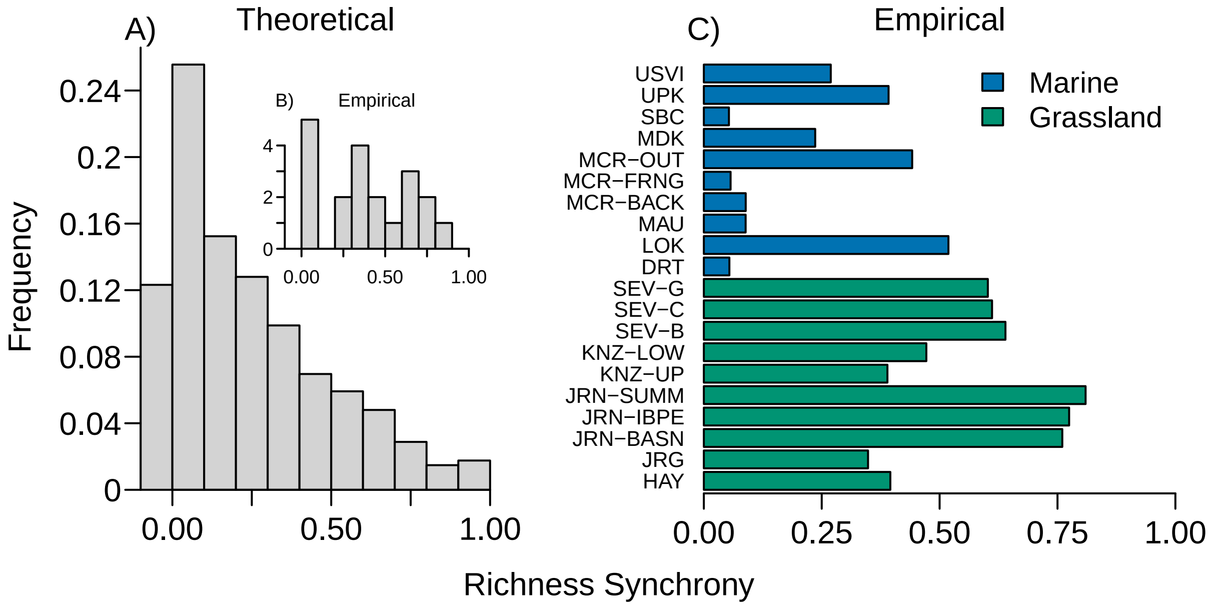


Figure S21: Spatial synchrony in species richness in (A) 2500 simulated and (B, C) 20 empirical metacommunities.

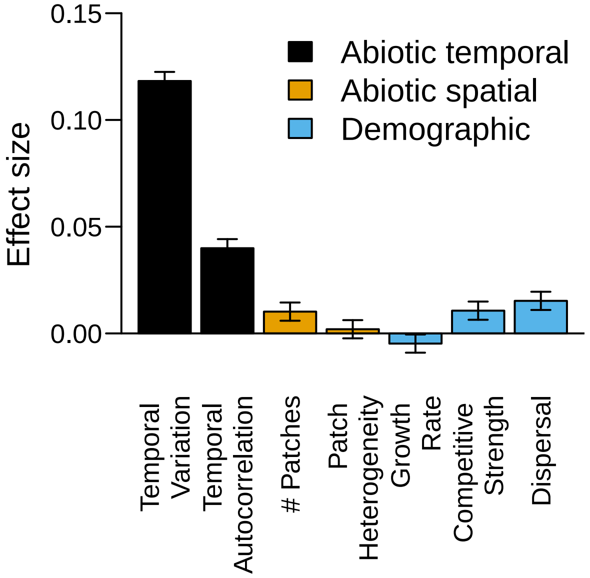


Figure S22: Effect sizes of variation in model parameters on the degree of spatial synchrony of richness in simulated metacommunities. Effect sizes are linear regression coefficients on standardized predictors. Error bars indicate 1 standard error.

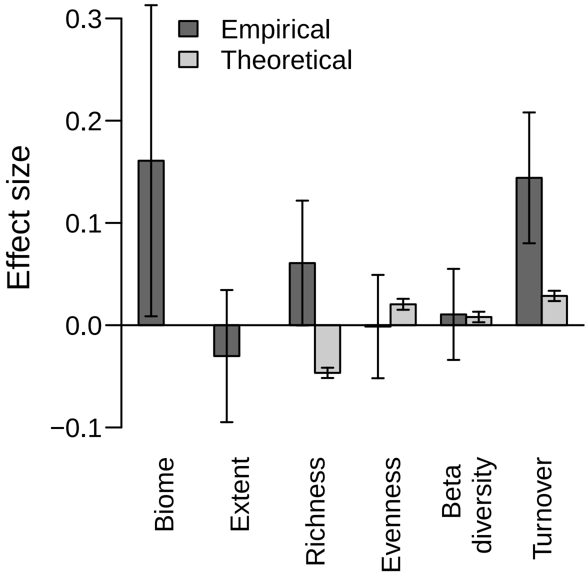


Figure S23: Effect sizes of variation in attributes of empirical and theoretical metacommunities on spatial synchrony of richness. Effect sizes are linear regression coefficients on standardized predictors. There is no direct analog of biome or extent in our theoretical simulations, so no bar is drawn. Error bars indicate 1 standard error.

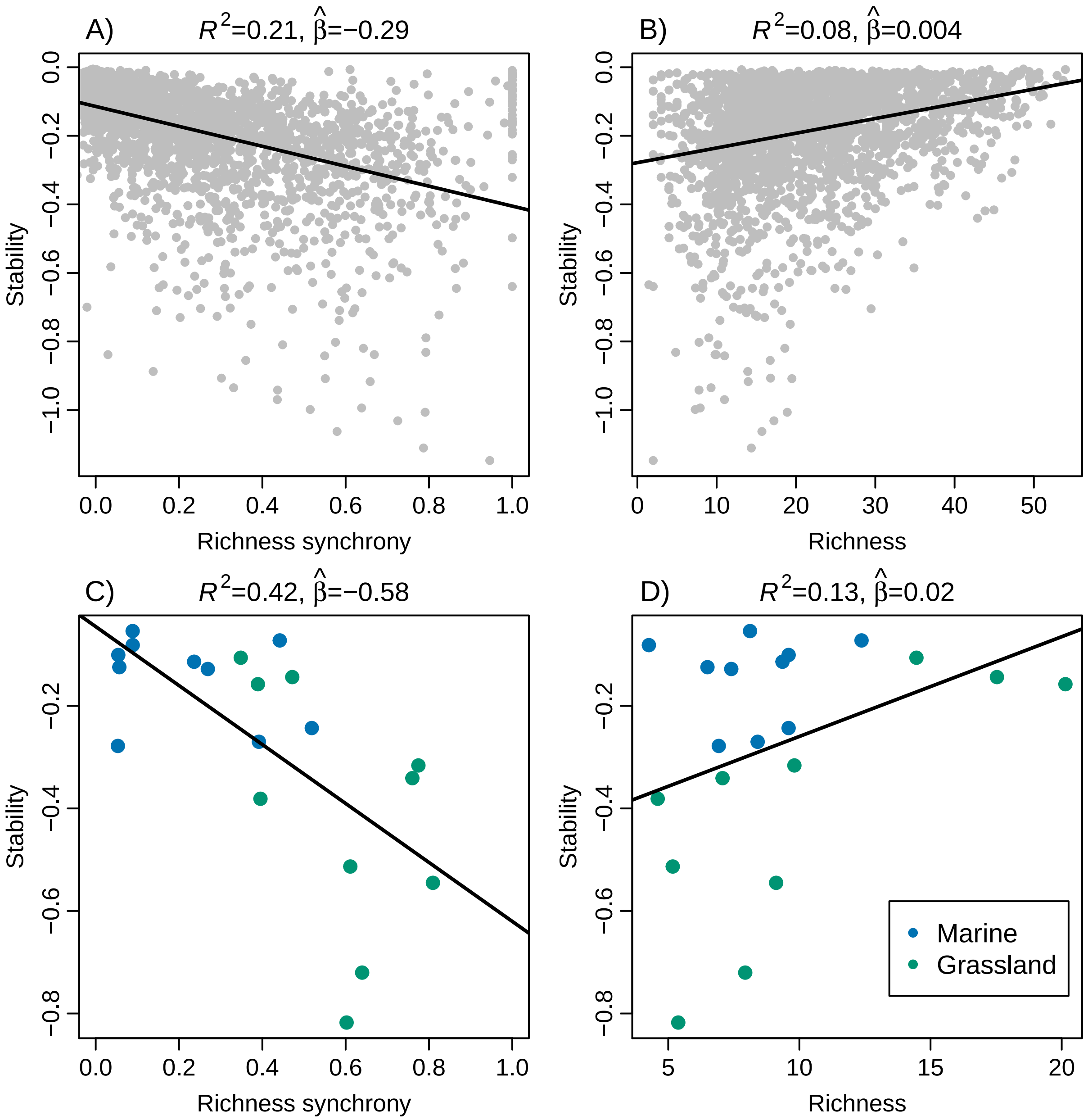


Figure S24: Richness synchrony is related to stability of ecosystem function in theoretical (A) and empirical (C) metacommunities, and more strongly so than species richness itself in both theoretical (B) and empirical (D) metacommunities. Stability is measured, for simulations, as the -1 × the coefficient of variation (CV) of total abundance, and for empirical datasets as that of variation of total biomass or total cover, depending on units of the underlying data.

## References

Adler, P. B., Tyburczy, W. R., and Lauenroth, W. K. (2007). Long-term mapped quadrats from kansas prairie: demographic information for herbaceous plants. *Ecology*, 88:2673.

Guest, J. R., Edmunds, P. J., Gates, R. D., Kuffner, I. B., Brown, E. K., Rodgers, K. S., Jokiel, P. L., Ruzicka, R. R., Colella, M. A., Miller, J., Atkinson, A., Feeley, M. W., and Rogers, C. S. (2018). Time-series coral-cover data from Hawaii, Florida, Mo'orea, and the Virgin Islands. U.S. Geological Survey data release. doi: 10.5066/F78W3C7W.

Harrer, S. L., Reed, D. C., Holbrook, S. J., and Miller, R. J. (2013). Patterns and controls of the dynamics of net primary production by understory macroalgal assemblage in giant kelp forests. *Journal of Phycology*, 49:248–257.

Hartnett, D. C. and Collins, S. L. (2016). PVC02 plant species composition on selected watersheds at konza prairie ver 7. Environmental Data Initiative. https://doi.org/10.6073/pasta/2871559b94adabdbd79c97d99905ee6d.

Hobbs, R. J. and Mooney, H. A. (1985). Community and population dynamics of serpentine annual grassland in relation to gopher disturbances. *Oecologia*, 67:342–351.

Moorea Coral Reef LTER and Carpenter, R. (2015). MCR LTER: Coral reef: Long-term population and community dynamics: Benthic algae and other community components, ongoing since 2005 ver 28. Environmental Data Initiative. https://doi.org.10.6073/pasta/79a6edbcf3aa2380d43deed778856416.

Moorea Coral Reef LTER and Edmunds, P. (2018). MCR LTER: Coral reef: Long-term population and community dynamics: Corals, ongoing since 2005 ver 35. Environmental Data Initiative. https://doi.org/10.6073/pasta/263faa48b520b7b2c964f158c184ef96.

Muldavin, E. and Moore, D. (2016). Core research site web seasonal biomass and seasonal and annual NPP data for the net primary production study at the sevilleta na-

tional wildlife refuge, new mexico (1999-present) ver 244946. Environmental Data Initiative. https://doi.org/10.6073/pasta/b913c188d07335b3e88fd585c1cd20b5.

Muldavin, E. H., Moore, D. I., Collins, S. L., Wetherill, K. R., and Lightfoot, D. C. (2008). Aboveground net primary production dynamics in a northern Chihuahuan Desert ecosystem. *Oecologia*, 155:123–132.

Peters, D. and Huenneke, L. (2015). NPP study: Quadrat field measurement data ver 64. Environmental Data Initiative. https://doi.org/10.6073/pasta/d65a72899a4294bc9e5e64158a6dbeae.

Rassweiler, A., Reed, D. C., Harrer, S. L., and Nelson, J. C. (2018). Improved estimates of net primary production, growth and standing crop of *Macrocystis pyrifera* in southern california. *Ecology*, 99:2132–2132.

Reed, D., Washburn, L., Rassweiler, A., Miller, R., Bell, T., and Harrer, S. (2016). Extreme warning challenges sentinel status of kelp forests as indicators of climate change. *Nature Communications*, 7:13757.

Rudgers, J. A., Chung, Y. A., Maurer, G. E., Moore, D. I., Muldavin, E. H., Litvak, M. E., and Collins, S. L. (2018). Climate sensitivity functions and net primary productivity: a framework for incorporating climate mean and variability. *Ecology*, 99:576–582.

Santa Barbara Coastal LTER and Reed, D. C. (2018). SBC LTER: Reef: Annual time series of biomass for kelp forest species, ongoing since 2000 ver 7. Environmental Data Initiative. https://doi.org/10.6073/pasta/d5fd133eb2fd5bea885577caaf433b30.
